# Exposure to childhood abuse is associated with human sperm DNA methylation

**DOI:** 10.1038/s41398-018-0252-1

**Published:** 2018-10-02

**Authors:** Andrea L. Roberts, Nicole Gladish, Evan Gatev, Meaghan J. Jones, Ying Chen, Julia L. MacIsaac, Shelley S. Tworoger, S. Bryn Austin, Cigdem Tanrikut, Jorge E. Chavarro, Andrea A. Baccarelli, Michael S. Kobor

**Affiliations:** 1 Department of Environmental Health, Harvard T.H. Chan School of Public Health, Boston, MA USA; 2 Department of Medical Genetics, Centre for Molecular Medicine and Therapeutics, and BC Children’s Hospital Research Institute, University of British Columbia, Vancouver, BC Canada; 3 Beedie School of Business, Simon Fraser University, Burnaby, BC Canada; 4 Department of Epidemiology, Harvard T.H. Chan School of Public Health, Boston, MA USA; 5 Channing Division of Network Medicine, Department of Medicine, Brigham and Women’s Hospital and Harvard Medical School, Boston, MA USA; 6 Division of Adolescent and Young Adult Medicine, Boston Children’s Hospital, Boston, MA USA; 7 Department of Urology, Massachusetts General Hospital and Harvard Medical School, Boston, MA USA; 8 Department of Nutrition, Harvard T.H. Chan School of Public Health, Boston, MA USA; 9 Laboratory of Environmental Precision Biosciences, Mailman School of Public Health, Columbia University, New York, NY USA; 10 Human Early Learning Partnership, School of Population and Public Health, University of British Columbia, Vancouver, BC Canada

## Abstract

Offspring of persons exposed to childhood abuse are at higher risk of neurodevelopmental and physical health disparities across the life course. Animal experiments have indicated that paternal environmental stressors can affect sperm DNA methylation and gene expression in an offspring. Childhood abuse has been associated with epigenetic marks in human blood, saliva, and brain tissue, with statistically significant methylation differences ranging widely. However, no studies have examined the association of childhood abuse with DNA methylation in gametes. We examined the association of childhood abuse with DNA methylation in human sperm. Combined physical, emotional, and sexual abuse in childhood was characterized as none, medium, or high. DNA methylation was assayed in 46 sperm samples from 34 men in a longitudinal non-clinical cohort using HumanMethylation450 BeadChips. We performed principal component analysis and examined the correlation of principal components with abuse exposure. Childhood abuse was associated with a component that captured 6.2% of total variance in DNA methylation (*p* < 0.05). Next, we investigated the regions differentially methylated by abuse exposure. We identified 12 DNA regions differentially methylated by childhood abuse, containing 64 probes and including sites on genes associated with neuronal function (*MAPT*, *CLU*), fat cell regulation (*PRDM16*), and immune function (*SDK1*). We examined adulthood health behaviors, mental health, and trauma exposure as potential mediators of an association between abuse and DNAm, and found that mental health and trauma exposure partly mediated the association. Finally, we constructed a parsimonious epigenetic marker for childhood abuse using a machine learning approach, which identified three probes that predicted high vs. no childhood abuse in 71% of participants. Our results suggested that childhood abuse is associated with sperm DNA methylation, which may have implications for offspring development. Larger samples are needed to identify with greater confidence specific genomic regions differentially methylated by childhood abuse.

## Introduction

Childhood abuse is associated with detriments in the mental and physical health of the victim across the life course^1–3^. Childhood abuse has also been associated with altered function of multiple biological systems^4–7^, with differences persisting into adulthood^8,9^. Changes in epigenetic marks have been proposed as a mechanism by which childhood abuse increases risk of neuropsychiatric and cardiometabolic disease^10,11^. Differences in epigenetic marks have been found in DNA methylation (DNAm) of blood^12,13^, saliva^14^, and brain tissue^15^ by experience of childhood abuse^16^. The association of childhood abuse with DNAm in gametes is of particular interest, both because the patterns of DNAm in gametes have been associated with fertility^17,18^ and the possibility that gamete DNAm may affect the healthy development of the offspring^19,20^.

In animal models, a variety of exposures have been shown to affect sperm DNAm, including nutritional status^21^, endocrine-disrupting hormones^22^, and other pollutants^23^. Animal experiments have also indicated that paternal stressors can affect DNAm^24^, gene expression^25,26^, and behavior^24,27^ in the offspring. In mice, exposure to social instability early in life leads to anxiety and defective social interactions, behaviors that are transmitted to three generations of offspring through the paternal line^28^. Transmission of paternal experiences of psychological trauma through gametes has also been documented^26^ and corresponds with alterations in paternal sperm DNAm^24^.

To our knowledge, no studies in humans have examined the effects of psychosocial stressors on sperm DNAm; however, psychological stress in humans has been associated with poorer semen quality, including lower motile sperm concentration, lower percentage of progressively motile sperm, and reduced lateral head displacement^29,30^. Evidence suggests that environmental exposures such as cigarette smoke^31^ and health status indicators, such as age^32^ and obesity^33,34^, are associated with sperm epigenetics in humans. Additionally, relevant to abuse in childhood, the pre-pubertal period has been identified as a potential window of sensitivity of the sperm epigenome to environmental influences^35^. Thus, it is possible that psychosocial stressors, including childhood abuse, affect the human sperm epigenome, including DNAm.

In the present study, we assessed the differences in genome-wide sperm DNAm in association with childhood abuse in a non-clinical longitudinal cohort of men. We calculated the principal components (PCs) of methylation values for all probes and examined the association of childhood abuse with PCs. DNAm sites typically function in concert with neighboring sites to affect gene expression^36^, thus it may be more meaningful to investigate DNAm within genomic regions as opposed to at individual sites. We therefore examined differentially methylated regions (DMRs) for association with childhood abuse. Finally, we used machine learning methods in order to identify sites predictive of childhood abuse from all sites and construct a parsimonious predictor of child abuse status. As childhood abuse has been associated with higher prevalence of adulthood health risk behaviors^37,38^, mental disorders^39,40^, and trauma exposure^41,42^, we conducted exploratory analyses to examine whether body mass index (BMI), smoking, depressive symptoms, posttraumatic stress symptoms, and trauma exposure accounted for a possible association of childhood abuse with sperm DNAm.

## Materials and methods

### Sample

The Growing Up Today Study (GUTS) is a US longitudinal cohort of 16,882 offspring of women participating in the Nurses’ Health Study II, enrolled in 1996 at ages 9–14 years and followed annually or biennially^43^. In 2010, male participants were asked whether they would be willing to donate a semen sample. Nearly two-thirds (64%) were willing. Age, BMI, and race did not differ between men willing and unwilling to donate. In 2012, we contacted 66 men to request a sample; 54 men (82%) returned the sample by mail. We further invited the first 28 men who returned the sample to send a second one; 24 men (86%) returned the second sample. Men were asked to abstain from ejaculation for at least 48 h prior to producing the sample by masturbation into a collection container (Thermo Scientific Nalgene Jars). Samples were shipped overnight, with four gel refrigerant packs surrounding the sample, to the Massachusetts General Hospital Fertility Center where sperm concentration and morphology were measured. Remaining semen was aliquoted and flash frozen in liquid nitrogen. Informed consent was obtained from all participants. The Institutional Review Board of Partners Healthcare approved this study.

We conducted DNAm assays on 48 samples from 34 men. Of these, 20 men contributed single samples, 12 men contributed two samples each, produced ~3 months apart, and two men’s samples were assayed twice as technical replicates, for a total of 48 samples assayed. We oversampled men who had been exposed to high levels of abuse, such that the samples that were assayed included 17 men exposed to high, 5 men to medium, and 12 men to no childhood abuse.

### Measures

Experiences of physical, emotional, and sexual abuse before age 18 were measured in 2007 when participants were aged 18–23 years. Physical and emotional abuse were measured with four items from the Childhood Trauma Questionnaire (CTQ), querying frequency that an adult in the family yelled, insulted, punished cruelly, and hit so hard that it left bruises^44^. Responses to the CTQ were summed^44^ and then divided into quartiles based on their distribution in the entire cohort (lowest quartile = 0 points, highest quartile = 3 points). Physical and emotional abuse were also measured with three items from the Conflict Tactics Scales (CTS), querying frequency that an adult in the family shoved; threatened to punch, kick, or hit with something; actually punched, kicked, or hit with something; or physically attacked^45^. Response options for the CTQ and the CTS ranged from “never” to “very often”. Responses to the CTS were skewed, with most respondents reporting none of these experiences. We therefore divided this scale into 0: lowest 50%, 1: next 25%, and 2: highest 25%.

Sexual abuse was queried in each time period with two questions regarding unwanted sexual experiences with an adult or older child (e.g., “Did an adult or an older child force you into any sexual activity by threatening you or hurting you in some way?”)^45^. Response options included: no; once; or > once.

To oversample men exposed to high levels of abuse, we created an overall measure of childhood abuse in three levels: none, moderate, and high. Men with “no abuse” (*N* = 12) were in the lowest category of both measures of physical and emotional abuse and had not experienced sexual abuse. Respondents with “high abuse” (*N* = 17) were either in the highest level of the CTS or the highest level of the CTQ, or had a mixture of elevated responses across both questionnaires. All or nearly all men in this group had experienced punishments that seemed cruel, were yelled and screamed at, and had hurtful and insulting things said to them. All had been shoved, grabbed, hit, or physically attacked in some other way, and most had also been threatened with violence. Two men in this group had been sexually abused. Five participants fell between the “no abuse” and “high abuse” groups and were considered to have experienced “medium” abuse. We also summed the CTQ, CTS, and sexual abuse measures to create a continuous measure of abuse severity (range, 0–7) and dichotomized participants as none-to-medium (0–2) vs. high abuse^3–7^.

### Covariates

We examined the characteristics of the semen sample, including ejaculate volume, sperm concentration, percent normal morphology, collection date, collection time, and abstinence interval, as well as characteristics of the participant, including age at collection, month of birth, and race/ethnicity as possible covariates. Additionally, we included information reported by the participants’ mothers, Nurses’ Health Study II cohort members, regarding her ancestry as well as participants’ childhood socioeconomic status, an index of family income, maternal social standing, and paternal education, reported in 1999–2001.

### Hypothesized mediators

Childhood abuse increases risk for adulthood health risk behaviors, mental disorders^46,47^, and trauma exposure^42^, factors that may explain an association of childhood abuse with adulthood sperm DNAm. We examined smoking, BMI (by self-report in 2010 and 2007), depressive symptoms (measured with the Center for Epidemiologic Studies Depression Scale-10^48^ in 2010), posttraumatic stress symptoms (measured with the 7-item Short Screening Scale for DSM-PTSD^49^ in 2007), and trauma exposure (measured in 2007 with 13 items adapted from the Brief Trauma Questionnaire^50^ e.g., physical assault, intimate partner violence, and serious illness) as potential mediators.

### DNAm assay

A differential lysis method involving a series of six washes was performed to separate sperm cells from epithelial and round cells (Supplemental Material). We then conducted DNAm assays with Infinium HumanMethylation450 (450 K) BeadChips (Illumina) using bisulfite-treated DNA (EZ-96 DNA Methylation kit, Zymo Research, Irvine, CA). These assays produce 485,577 data points encompassing 482,421 CpG sites and 3091 CpN sites. Raw intensity scores were color corrected and background was subtracted using GenomeStudio Software (Illumina). Methylation *β* value for each probe represents a continuous ratio between 0 (0% methylated) and 1 (100%). Probes were excluded from further analysis if they had a detection *p*-value < 0.01 (*n* = 2144 probes) or if > 5% of samples were missing a *β* value (*n* = 12,353 probes). Probes which bound in silico to the X and Y chromosome in addition to the specified targets were excluded^51^, leaving *N* = 439,746 probes available for subsequent analysis. Inter-sample normalization was performed using quantile normalization^52^. To account for the two probe types on the Illumina BeadChip, normalization was performed using subset-quantile within array normalization (SWAN)^53^. To determine if there were batch effects, PCA was performed on the normalized data followed by Spearman’s correlations of the PCs with all technical variables. A slight batch effect associated with chip number and position was removed using empirical Bayes methods (R package SVA, ComBat function, Supplementary Figure S1)^54^.

To evaluate the purity of our washed sperm samples, we compared DNAm in our sample with DNAm from an independent study of contaminated and purified sperm samples (Gene Expression Omnibus (GEO)^55^ GSE108058, Supplementary Figure S2). We merged the GEO dataset with our own data and performed PCA. The vast majority of variation in methylation is associated with tissue heterogeneity, therefore the first few PCs should be correlated with the purity of the semen samples. Plotting PC1 against PC2 (for visualization purposes), our samples clustered with the pure semen, providing evidence that we had successfully purified our samples. We additionally examined the methylation status of two imprinting control regions (*HYMAI* and *GNAS-AS*). These regions are paternally expressed, and therefore we would anticipate that these regions would be fully unmethylated if our samples contained purified haploid gametes (as opposed to hemi-methylated in somatic tissue). We calculated the median DNAm *β* value for each probe underlying these regions (130 probes) for each sample in our study. The vast majority of samples had median *β* < 0.05, suggesting good purity (Supplementary Table S1, Supplementary Figure S3).

### Analyses

To characterize the study sample, we compared age, race, and abuse exposure of study participants with all GUTS men. Next, for study participants, we calculated prevalence for categorical variables and mean for continuous variables for covariates by childhood abuse status.

#### Principal components analysis

To investigate whether childhood abuse and our covariates were associated with variation in DNAm, we conducted PCA with all probes (*N* = 439,746) using one randomly selected sample per subject. PCA reduces the dimensionality of the data by identifying orthogonal components from methylation values of all individual probes, with PC1 explaining the most variance. We examined the association of both the continuous and categorical childhood abuse variables and the covariates with centered PCs, using one-way ANOVAs for ordinal and categorical variables and Spearman’s correlations for continuous variables. For PCs that were statistically significantly associated with childhood abuse, we investigated which specific probes contributed most to the PC by first identifying individual probes with the largest PC score (the 1% of probes with the largest positive scores and 1% with the largest negative scores) and then, to increase the likelihood of biological relevance, selected only probes with methylation Δ*β* ≥ 5%, where $\Delta \beta = \bar \beta$_high abuse_ –$\bar \beta$_no abuse_. *P*-values were not adjusted for multiple testing, as this was an exploratory analysis to determine associations with DNAm.

#### DMRs analysis

We next investigated whether childhood abuse was associated with patterns of DNAm in spatially clustered probes. We investigated DMRs by childhood abuse exposure using the R package DMRcate^56^ (Bioconductor, http://www.bioconductor.org), using the same randomly selected sample per subject used in the PCA. DMRcate first assesses the association of the exposure (childhood abuse) with methylation at each individual CpG site, then groups the probes into DMRs based on the similarity of effect size and directionality with distances of ≤ 1000 bp between them. DMRs are then corrected for multiple testing by calculating the false discovery rate (FDR) for each DMR. DMRs that do not meet an FDR ≤ 0.05 and a fold change ≥ 0.05 are dropped. We considered regions to be DMRs if they were statistically significant at an FDR ≤ 0.05, contained ≥ 3 probes, and had a difference in DNAm *β* (Δ*β*) ≥ 5%, where Δ*β* = $\bar \beta$_high abuse_ –$\bar \beta$_no abuse_. We conducted these analyses with the ordinal childhood abuse variable to reduce the effects of outliers, then checked that results were similar in analyses using the continuous childhood abuse variable. We verified our findings by replacing the sample used in the primary analyses with the replicate sample from each man who contributed two samples (*N* = 12) and re-running the DMR analyses using original samples from 22 men and replicate samples from 12 men. Finally, in sites located in identified DMRs, we calculated the interclass correlation coefficient (ICC) between the first and second sample in DNAm *β* values.

To examine the concordance of our two methods of identifying probes differentially methylated by childhood abuse, we compared the overlap in probes identified using PCA and probes identified in DMR analysis.

#### Machine learning analysis

Finally, we used machine learning to identify sites predictive of childhood abuse and: (1) compare them with the sites identified in the DMR analysis and (2) construct a parsimonious predictor of child abuse status. We fit a penalized linear regression (“elastic net”) to select informative probes from the set of all probes using the dichotomized childhood abuse variable (none/medium vs. high abuse, mixing parameter α set to 0.5, the default). The penalized regression begins by fitting a single linear model including all probes, then selects a subset of relevant probes by shrinking the linear coefficients and setting to zero coefficients below a given threshold^57^. The selected probes are those with non-zero coefficients. We estimated the penalty parameter λ with tenfold cross-validation and set it to 0.095. We applied the resulting predictor to three independent datasets (Gene Expression Omnibus^55^ GSE108058, GSE102970^58^, and GSE64096^59^**)** to ascertain whether the prevalence of abuse estimated with this predictor was approximately the same as the prevalence in the whole GUTS cohort (high abuse prevalence = 28.8%). As no datasets of sperm DNAm were available with childhood abuse measured, we could not test its ability to predict abuse status.

#### Pyrosequencing methylation confirmation

To confirm findings from the 450 K array, we performed pyrosequencing with bisulfite-converted DNA. We selected five sites for confirmation, prioritizing sites within DMRs and sites with low FDR. We calculated Spearman correlations between *β* values obtained from pyrosequencing and the 450 K array and performed linear regression to ascertain the association of pyrosequencing *β* values with childhood abuse.

#### Exploratory mediation analysis

To examine whether adulthood health risk factors might explain a possible association between childhood abuse and DNAm, we conducted two analyses. First, we examined whether these risk factors loaded on DNAm PCs, using one-way ANOVAs for ordinal variables and Spearman’s correlations for continuous variables. Next, we examined probes identified in DMR analyses. For each probe in a childhood abuse DMR, we compared the association of childhood abuse with DNAm in linear models adjusted only for age and semen volume (base model) and in models further adjusted for: (1) health risk behaviors (smoking and BMI); (2) mental health (depressive and posttraumatic stress symptoms); and (3) trauma exposure. We calculated % mediation as: [(*β*_child abuse, base model_–*β*_child abuse, adjusted model_)/*β*_child abuse, base model_]*100 for each probe and calculated the mean mediation across all probes within each DMR for each set of hypothesized mediators. We did not include all hypothesized mediators in a single model to avoid overfitting.

#### Probes associated with childhood abuse in prior studies

We examined the association of 1667 probes previously identified as associated with childhood abuse^11,14–16^. We considered probes with FDR < 0.05 as statistically significant, accounting for multiple testing within this set of 1667 probes.

#### Code availability

Code is available at GitHub^60^.

## Results

Study participants were similar to all GUTS participants in age (participants, mean = 25.7 years, range = 23–29 years; GUTS, mean = 25.8 years, range = 23–31 years) and race/ethnicity (participants, 91.2% white; GUTS, 93.2% white), and had a higher prevalence of exposure to high levels of childhood abuse (participants, no abuse = 35.3%, high = 50%; GUTS, no abuse = 26.3%, high = 28.8%). Characteristics of study participants and semen samples were similar across levels of exposure to child abuse (all *p* > 0.05, Table 1).

**Table 1:** Participant and semen sample characteristics by experience of childhood abuse (*N* = 34)

		Experience of childhood abuse		
		None	Medium	High
		(*N* = 12)	(*N* = 5)	(*N* = 17)
*Covariates*				
Age, years	Mean (range)	26.3 (24 –28)	25.4 (23–27)	25.2 (23–29)
*Race/ethnicity*				
White	% (*N*)	91.7 (11)	100.0 (5)	88.2 (15)
Nonwhite	% (*N*)	8.3 (1)	0.0 (0)	11.8 (2)
Maternal ancestry				
Scandinavian	% (*N*)	0 (0)	20.0 (1)	11.8 (2)
Southern European	% (*N*)	41.7 (5)	20.0 (1)	17.7 (3)
Other Caucasian	% (*N*)	83.3 (10)	60.0 (3)	70.6 (12)
Hispanic	% (*N*)	0 (0)	0 (0)	5.9 (1)
Childhood socioeconomic status	Mean (SD)	7.3 (1.5)	7.6 (1.5)	7.0 (1.9)
Semen volume, ml	Mean (SD)	2.5 (1.3)	3.9 (1.7)	2.7 (1.8)
Sperm concentration, M/ml	Mean (SD)	56.1 (26.8)	56.2 (18.7)	53.1 (29.4)
Normal sperm morphology	% (SD)	7.8 (3.5)	7.8 (5.5)	6.5 (2.9)
Collection time, morning	% (*N*)	91.7 (11)	60.0 (3)	64.7 (11)
Abstinence time, hours	Mean (SD)	92.8 (17.6)	97.4 (21.6)	83.0 (11.7)
*Hypothesized mediators*				
Smoking				
Current	% (*N*)	8.3 (1)	0 (0)	23.5 (4)
Past	% (*N*)	16.7 (2)	20.0 (1)	5.9 (1)
BMI	Mean (SD)	24.0 (3.2)	24.1 (2.9)	24.3 (4.7)
Depressive symptoms	Mean (SD)	5.7 (4.7)	5.5 (5.6)	7.7 (5.0)
Posttraumatic stress symptoms	Mean (SD)	1.2 (0.3)	1.3 (0.5)	2.0 (1.2)
Traumatic events	Mean (SD)	0.2 (0.4)	1.6 (1.5)	1.4 (1.7)

SD, standard deviation

Maternal ancestry by maternal self-report in 1989. Ancestry percentages do not sum to 100, as women could endorse more than one ancestry. No mothers reported African, American, Asian, or “other” ancestry. Childhood socioeconomic status is an index of family income in 2001, paternal educational attainment in 1999, and maternal perceived social standing in the US in 2001. Normal sperm morphology ascertained according to World Health Organization (2010)^94^

### Principal components analysis

**Fig. 1:**
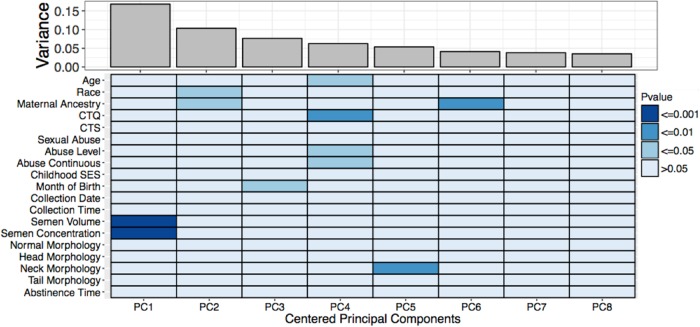
Principal component 4 (PC4) was associated with childhood abuse exposure (one sample per participant, *N* = 34). PC4, representing 6.24% of the variance present in the methylation data, was significantly correlated (*p* *<* 0.05) with childhood abuse exposure. Darker regions signify stronger correlations between variables and principal components (*N* probes = 439,746). Normal sperm morphology is characterized, beginning at the head and moving toward the tail. Thus, “head morphology” is the % of sperm in a sample with normal heads, “neck morphology” is the % with normal heads and necks, and “tail morphology” is % with normal head, neck, and tail. Abstinence time is the time between the sperm donation and the most recent preceding ejaculation. PC principal component, CTQ Childhood Trauma Questionnaire, CTS Conflict Tactic Scale

PC4 was correlated with childhood abuse (Spearman’s correlation *p* ≤ 0.05) and explained 6.2% of the variation in DNAm (Fig. 1). Participant’s age was also correlated with PC4 and adjusted in DMR analyses. To identify probes that were both strongly associated with PC4 and were related to childhood abuse exposure, we selected probes with the largest PC4 scores (*N* = 8795) and then from these selected probes with DNAm Δ*β* ≥ 5% between high and no abuse, resulting in over 1000 probes (*N* = 1137, Supplementary Table S2). The two men who had experienced sexual abuse were not outliers among men who experienced abuse (Supplementary Figure S4).

### DMRs analysis

**Fig. 2:**
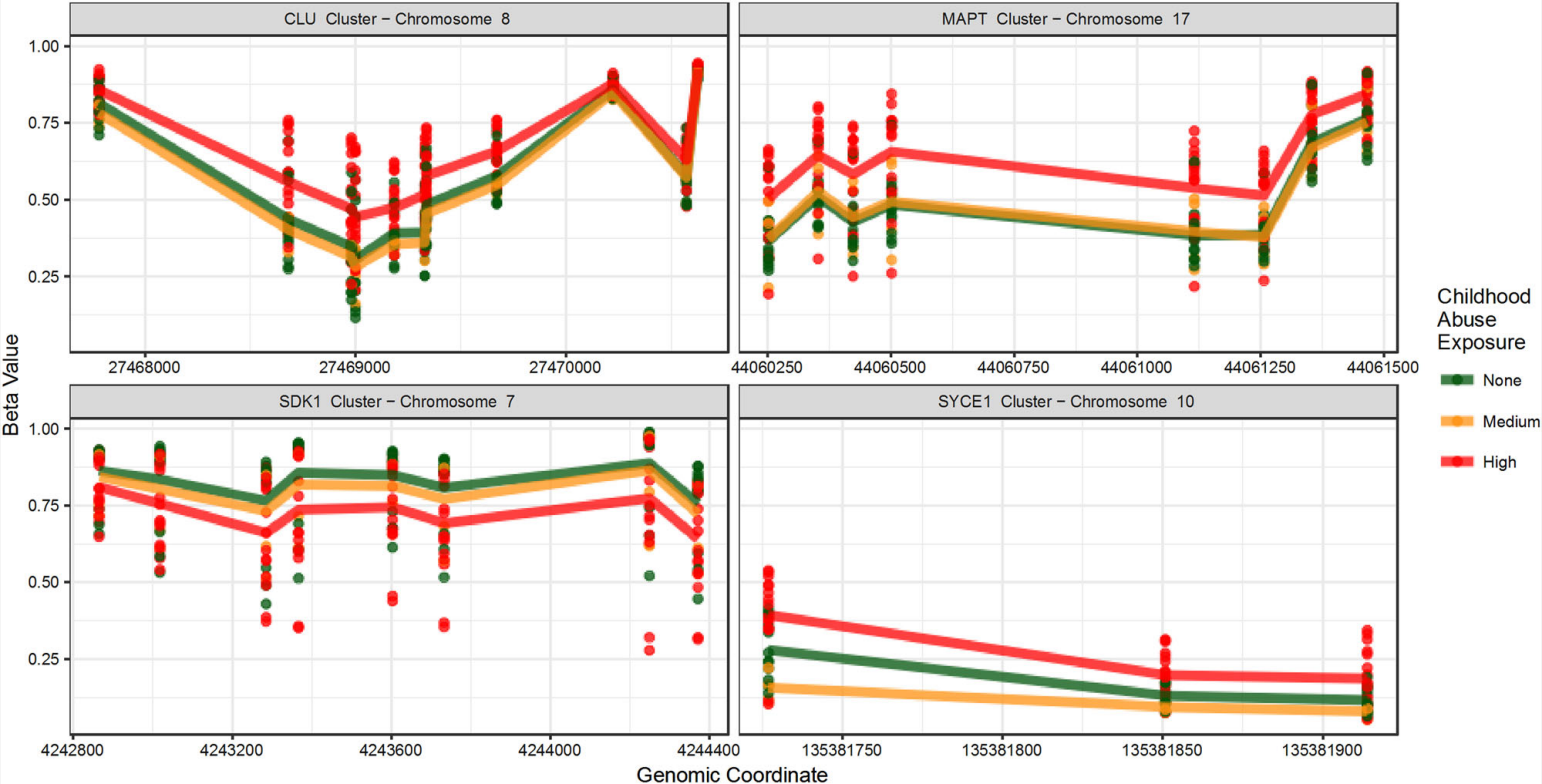
Four genomic regions differentially methylated by childhood abuse. Differentially methylated regions (DMRs) were defined as regions that differed statistically by abuse exposure at an FDR ≤ 0.05, had a mean Δ*β* ≥ 5% across probes, and were confirmed using replicates. The “*CLU* cluster” includes the 5’ UTR transcription start site and part of the gene body spanning 2.8 kb. The “*MAPT* cluster” is located in the gene body and spans 1.2 kb. The “*SDK1* cluster” is located in the gene body and spans 1.5 kb. The “*SYCE1* cluster” is located in the 5’ UTR and spans 200 bp

We identified 13 DMRs meeting our criteria: (1) FDR ≤ 0.05; (2) mean Δ*β* ≥ 5%; and (3) contained ≥ 3 probes. Of these 13 DMRs, 12 met these three criteria in analyses using original samples from 22 men and replicate samples from 12 men (*N* = 34). These 12 DMRs contained 64 probes (Table 2, Fig. 2, and Supplementary Figure S5). Three DMRs were located in enhancers, two were located in transcription start sites, six were located in CpG islands, and three were located in gene bodies (Supplementary Table S3). The ICC between replicate samples (*N* = 12) for the 63 CpG sites comprising these 12 DMRs was greater than 0.7 for 90% of sites (Supplementary Figure S6). Results were similar with childhood abuse coded as a continuous variable.

**Table 2:** Differentially methylated regions (DMRs) associated with childhood abuse exposure

Cluster name	Number of significant probes	*p*-value	FDR	Average ∆*β*	Max ∆*β*
ARL17A	3	1.54E-10	2.43E-07	−0.29	−0.35
MAPT	8	7.66E-10	7.99E-07	0.132	0.173
CLU	11	9.82E-05	1.04E-02	0.08	0.139
LRRK1	3	1.03E-17	1.19E-13	0.103	0.12
PRDM16	7	4.13E-05	6.95E-03	0.094	0.148
TCERG1L	3	1.60E-04	2.26E-02	0.131	0.147
CFAP46	5	2.09E-04	2.61E-02	−0.108	−0.122
MIR5093	4	2.52E-07	1.49E-04	0.108	0.128
TAF1B	3	6.47E-05	1.19E-02	0.148	0.194
DLL1	5	4.13E-05	8.52E-03	0.115	0.135
SYCE1	3	1.14E-09	1.31E-06	0.083	0.114
NDFUA10	3	1.60E-06	6.80E-04	0.119	0.138
SDK1	8	1.60E-04	1.93E-02	−0.091	−0.12

Statistically significant DMRs were discovered using DMRcate (FDR ≤ 0.05), had a mean Δ*β* ≥ 5%, and were verified using replicates. *P*-value, FDR, and mean Δ*β* for each DMR are the mean across all probes within the DMR. Δ*β* values were calculated as the difference between the mean *β* for high and no childhood abuse.

Sites identified in the DMR analysis overlapped considerably with sites identified in the PC analysis. Thirty-five of the 63 CpG sites in the DMRs were among the sites loading most strongly on PC4.

### Pyrosequencing

**Fig. 3:**
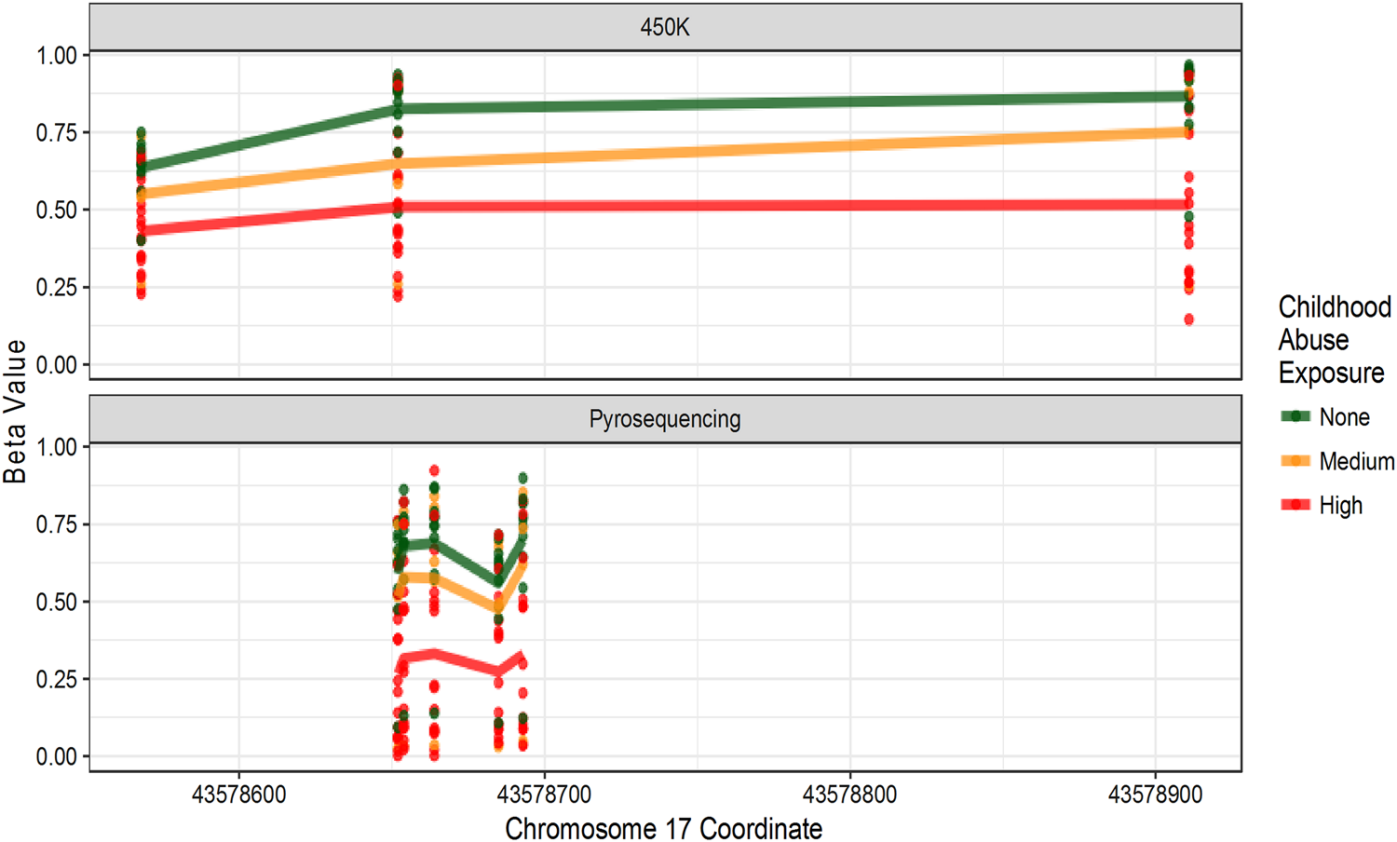
Additional sites measured during pyrosequencing of “*ARL17A* cluster” correlated significantly with 450 K sites in relation to childhood abuse. The “*ARL17A* cluster” found using DMRcate is located 30 kb away from *ARL17A* and spans 344 bp. The 450K methylation measurements of site cg04703951 (top panel) was confirmed using pyrosequencing techniques (bottom panel). The pyrosequencing assay measured DNAm at four additional sites not represented on the 450 K array (bottom panel). SD standard deviation, DMR differentially methylated region, FDR false discovery rate

For pyrosequencing confirmation of 450 K array results, we selected four CpG sites contained in childhood abuse DMRs: the *ARL17A* cluster (cg04703951), the *MAPT* cluster (cg00438222) and the *LRRK1* cluster (cg09926099 and cg00293616), and one additional site (cg08780220) based on its low FDR. All sites had significantly high correlations between measurements obtained by 450 K and pyrosequencing (Spearman’s rank ρ ≥ 0.74, *p* ≤ 4.0 × 10^–7^, Supplementary Figure S7), and were significantly associated with childhood abuse in linear regressions after correction for multiple testing (Supplementary Figure S8, Supplementary Table S4). The pyrosequencing assay for cg04703951 additionally measured DNAm at four CpG sites not represented on the 450 K array. These four additional sites were highly correlated with neighboring sites measured on the 450 K array (ρ ≥ 0.88) and differed significantly by childhood abuse (*p* ≤ 3.9 × 10^–8^, Fig. 3, Supplementary Table S4).

### Machine learning analyses

The machine learning approach identified three probes (cg02622647, cg04703951, and cg17369694) as most useful for classifying participants as none or medium vs. high abuse exposure. These probes correctly classified 71% of participants (12 true positives, five false positives, 15 true negatives, and two false negatives). Two of these three probes were also identified in the DMR and PC analyses (cg02622647 and cg04703951, *ARL17A* cluster), showing the concordance of these methods. In three independent datasets, NCBI GEO accession GSE108058, GSE102970^58^, and GSE64096^59^, this three-probe predictor predicted abuse prevalence of 30%, 35%, and 25%, respectively, similar to the 29% found in the GUTS cohort.

### Mediation analyses

None of our hypothesized mediators was associated with PC4, the PC that was significantly associated with childhood abuse. For probes in child abuse DMRs, the association of childhood abuse with DNAm was somewhat attenuated in models also including depressive and posttraumatic stress symptoms (two of 12 DMRs, mean mediation = 11.2 and 13.6%) and in models including lifetime trauma exposure (four of 12 DMRs, mean mediation range = 14.0–23.7%), but not in models including smoking and BMI (mean mediation < 5.7% for all DMRs). The association of childhood abuse with DNAm was somewhat *stronger* after adjustment for mental health in two DMRs (*DLL1* and *SYCE1*) and after adjustment for lifetime trauma in three DMRs (*MAPT*, *DLL1*, and *NDFUA10*, Supplementary Tables S5-S7).

We did not find a statistically significant association of childhood abuse with any of the candidate probes identified in prior studies of childhood abuse^11,14–16^.

## Discussion

Childhood abuse has been associated with alterations to multiple biological systems in adulthood^9^, and several studies have found differences in DNAm in somatic tissue by childhood abuse^16^. We examined whether childhood abuse was associated with sperm DNAm in adulthood and found evidence that sperm DNAm varies by experiences of childhood abuse. The three approaches we used to identify differences in DNAm associated with childhood abuse, PCA, DMR analysis, and machine learning, found significantly overlapping sites. Moreover, pyrosequencing assays identified additional sites proximate to and correlated with sites measured by the 450 K array that were also differentially methylated by childhood abuse. Together these findings suggest that our results reflect differences in DNAm associated with abuse.

Several DMRs we identified were located within genes, although it is unknown whether these specific sites are associated with the expression of the gene in spermatogonia or, if so, whether the Δ*β* we found by abuse level has biological significance. Clusterin (CLU) is an extracellular molecular chaperone expressed in the brain and embryonic tissues that responds to stress conditions and has been implicated in neurodegenerative disorders, including Alzheimer’s and Parkinson’s disease^61^. Additionally, clusterin RNA transcripts pass from human sperm to the oocyte at fertilization^62^. MAPT is hypothesized to be involved in neuronal migration and in establishing neuronal polarity^63^ and has been implicated in neuroticism^64^ and neurodegenerative disorders. PRDM16 is a transcriptional regulator involved in the regulation of fat cells^65,66^. *SDK1* encodes a protein in the immunoglobulin superfamily^63^. Thus, DMRs were found on genes coding for proteins with a variety of functions, consistent with the documented effects of childhood abuse on the brain, body weight, and immune system. The DMRs we found did not overlap with prior DMRs identified in a study of paternal sperm and offspring symptoms of autism spectrum disorder^67^ nor with probes in brain tissue, saliva, and peripheral blood identified in prior studies of childhood maltreatment^11,14–16^.

We found that higher trauma exposure and higher prevalence of depressive and posttraumatic stress symptoms in men who experienced childhood abuse compared with men who did not accounted for some of the association between childhood abuse and sperm DNAm in five DMRs. Childhood abuse and other types of traumatic events have common biological effects, e.g., on the HPA-axis^68,69^ and systemic inflammation^70^, thus it is plausible that abuse and other trauma types share effects on DNAm as well^71,72^. However, the association of childhood abuse with DNAm was also *stronger* in five DMRs after further adjustment for mental health and lifetime trauma. Taken together, these mediation results are also consistent with chance.

Our findings should be considered in light of important limitations. First, our sample size was small. Therefore, our identification of DMRs associated with childhood abuse should be interpreted cautiously and be used primarily as a starting point for further research. Due to our small sample size, our examination of adulthood sequelae of childhood abuse that might mediate a relation between abuse and DNAm must be considered exploratory. Second, our sample was predominantly white, thus, our findings may not apply to men of other races.

Animal studies have indicated that psychosocial stressors can affect both epigenetic patterns in sperm and offspring phenotype. Male mice conditioned to odor-related fear exhibited differences in sperm DNAm at a locus related to the odor receptor. These fear-conditioned mice produced two generations of offspring with the same odor-related fear response as well as corresponding alterations to neuronal structures, results that were robust to cross-fostering and in vitro fertilization^24^. Mice exposed to chronic stressors showed greater concentration of nine sperm micro RNAs (miRNAs) and HPA-axis alterations in offspring^26^. Injection of these nine miRNAs in zygotes produced similarly altered HPA-axis function, suggesting a causal role for the miRNAs in offspring biology^73^. In another experiment, mice exposed early in life to unpredictable maternal separation had altered patterns of small noncoding RNAs (sncRNAs) in sperm and had offspring with behavioral differences compared with control offspring. Injection of RNA from sperm into fertilized oocytes reproduced these behavioral differences^27^. Thus, robust experiments have indicated that stressors may affect murine sperm epigenetics, including DNAm, and offspring biology.

Evidence that psychosocial stressors affect human sperm epigenetics remains limited. To our knowledge, our study is the first to document an association of psychosocial stressors and sperm epigenetics in humans. Indirect evidence that stressors could affect sperm epigenetics in humans is suggested by studies finding reduced sperm quality in men exposed to psychosocial stressors^29,74,75^, as well as the association of other kinds of environmental exposures with human sperm epigenetic patterns^31,34,76–78^.

While most mammalian paternal epigenetic marks are erased at fertilization and again during preimplantation development^79^, some loci are resistant to demethylation^80^, and these preserved epigenetic marks may be biologically important. Several pieces of evidence suggest that human sperm epigenetics influences both fertility and embryogenesis: (1) sperm cells are transcriptionally silent yet have epigenetic marks characteristic of transcription^81,82^; (2) sperm chromatin has patterns of histone modifications at loci related to embryo development^81,82^; (3) sperm mRNA, produced prior to transcriptional arrest, are transferred to the oocyte^62^; and (4) sperm epigenetic marks are associated with fertility^82^.

Childhood abuse greatly precedes the time period in which the ejaculated sperm were dividing and maturing, thus could not directly affect sperm DNAm at this stage. Instead, childhood exposures may affect the epigenome of spermatogonia, which then gets propagated during spermatogenesis in adulthood^35,83^. Additionally, our results suggest that childhood abuse may lead to adulthood exposures that affect the sperm epigenome during spermatogenesis^23^. Regardless of their origin, it is tempting to speculate that these DNAm marks are somehow propagated to the offspring. However, research in human developmental biology has not yet provided strong evidence for this possibility^82^. Moreover, we note that offspring inherits the material from a single sperm, for which each CpG site is either methylated or unmethylated. If differences in DNAm associated with child abuse render affected sperm less likely to fertilize an egg, then the potential impact of these changes on offspring would likewise be reduced. Studies in humans have documented adverse neurodevelopmental outcomes in offspring of persons exposed to severe psychosocial stressors, in particular, to childhood abuse^38,84–91^. The hypothesis that the experience of stress may affect offspring through the parental epigenome has been raised as a potential mechanism for these associations^92,93^. While this possibility is intriguing, molecular evidence from human germ cells remains sparse. Our results recommend further consideration of this promising hypothesis.

## Electronic supplementary material

Preparation of samples

Supplemental Material
