# Supplementary material for: Exposure to childhood abuse is associated with human sperm DNA methylation
Source: Transl Psychiatry. 2018 Oct 2;8:194. doi: 10.1038/s41398-018-0252-1 (PMC6168447; doi:10.1038/s41398-018-0252-1)
Supplement: Supplementary file 1 — Preparation of samples [file 41398_2018_252_MOESM1_ESM.docx]

**Supplemental Material: Preparation of Sperm DNA for spPCR by Differential Lysis**

To separate sperm from epithelial and round cells in raw seman samples. Pure sperm cell DNA will be purified using the QIAGEN DNeasy Blood & Tissue Kit. Sterile techniques and reagents utilized.

Lysis and DNA Extraction Procedure:

1. Make 1xSSC: Dilute 4mL of 5xSSC in 16mL distilled water.
2. Make 0.2xSSC: Dilute 1 mL of 5xSSC in 4mL distilled water.
3. Prepare buffer X2 (25mL) if needed, was already made in Differential Lysis #14

| **Components** | **[Stock]** | **[Final]** | **mL to add** |
| --- | --- | --- | --- |
| Tris-Cl, pH 8.0 | 1M | 0.02M (20mM) | 0.5 |
| EDTA | 0.5M | 0.02M (20mM) | 1 |
| NaCl | 3M | 0.2M (200mM) | 1.67 |
| SDS | 20% | 4% | 5 |
| Distilled Water | - | - | 16.83 |

1. Remove raw semen samples from freezer to thaw.
2. Prepare 1xSSC + 1% SDS master mix:

| **Components** | **[Stock]** | **[Final]** | **mL to add for 1mL Sample** | **x4.4 Samples** |
| --- | --- | --- | --- | --- |
| SSC | 1x | 1x | 2.85 | 12.54mL |
| SDS | 20% | 1% | 0.15 | 0.66mL |
| Total Volume | - | - | 3.0 | - |

1. Finishing preparing Buffer X2 immediately before use:

| **Components** | **[Stock]** | **[Final]** | $\text{μL}$ **to add for 1 sample** | **X4.4 samples** |
| --- | --- | --- | --- | --- |
| DTT | 1M | 80mM | 16 | 70.4$\text{μL}$ |
| QIAGEN Proteinase K | - | 12.5$\text{ }\text{μL}$ per mL | 2.5 | 11.0$\text{ }\text{μL}$ |
| Buffer X2 | - | - | 181.5 | 798.6$\text{ }\text{μL}$ |
| Total Volume | - | - | 200 | - |

1. **Combine** each semen aliquot with 1mL 1xSSC + 1%SDS in Corex centrifuge tube. Pipet 3x.
2. **Pellet** sperm by spinning down at 10,000g for 10mins or until pellet is visible. Remove supernatant and avoid disturbing the pellet.
3. **Rinse** pellet with 1mL 1xSSC, 1%SDS, pipette to resuspend. Spin at 10,000g for 5mins. Remove supernatant and avoid disturbing the pellet. Repeat this step.
4. **Rinse** pellet in 500$\text{μL}$ 0.2x SSC, spin at 10,000g for 5 mins, and remove supernatant.
5. **Resuspend** sperm pellet in 500$\text{ }\text{μL}\text{ }$0.2x SSC and transfer to a labelled microcentrifuge tube.
6. **Add** 500$\text{ }\text{μL}$ 0.2x SSC to Corex tube, vortex 10s to collect any sperm adhering to the tube and transfer to the same microcentrifuge tube.
7. **Centrifuge** at 20,000g for 3mins, or until pellet is visible, and remove the supernatant.
8. **Resuspend** sperm in 200$\text{ }\text{μL}$ Buffer X2. Incubate in 56$℃$ shaking water bath at 100rpm until the sample is dissolved (at least 2 hours).
9. **Add** 400$\text{ }\text{μL}$ Buffer AL and ethanol mix to each sample. Vortex immediately.
10. Premix 1000$\text{ }\text{μL}$ Buffer AL and 1000$\text{ }\text{μL}$ ethanol (96-100%).
11. **Pipet** each mixture from step 12 (including any precipitate) into a DNeasy Mini spin column placed in a 2mL collection tube (provided). Centrifuge at 6,000g for 1min.
12. **Reload** the eluded flow-through into each column. Centrifuge again at 6,000g for 1min.
13. **Place** the DNeasy Mini spin column in a new 2mL collection tube. Add 500$\text{ }\text{μL}$ Buffer AW1 and centrifuge for 1min at 6,000g. Discard flow-through and collection tube.
14. **Place** the DNeasy Mini spin column in a new 2mL collection tube (provided). Add 500$\text{ }\text{μL}$ Buffer AW2 and centrifuge for 3min at 20,000g. Discard flow-through and centrifuge for 1min at 20,000g to dry the membrane. Discard flow-through and collection tube.
15. **Place** the column in a 1.5mL microcentrifuge tube and pipet 100$\text{ }\text{μL}$ Buffer AE onto the membrane. Incubate at room temp for 15mins, centrifuge at 6,000g for 1min to elute DNA.
16. **Repeat** step 18 with 50$\text{ }\text{μL}$ in each column, eluting into the same microfuge tube.
