# Supplementary material for: Exposure to childhood abuse is associated with human sperm DNA methylation
Source: Transl Psychiatry. 2018 Oct 2;8:194. doi: 10.1038/s41398-018-0252-1 (PMC6168447; doi:10.1038/s41398-018-0252-1)
Supplement: Supplementary file 2 — Supplemental Material [file 41398_2018_252_MOESM2_ESM.pptx]

## Slide 1
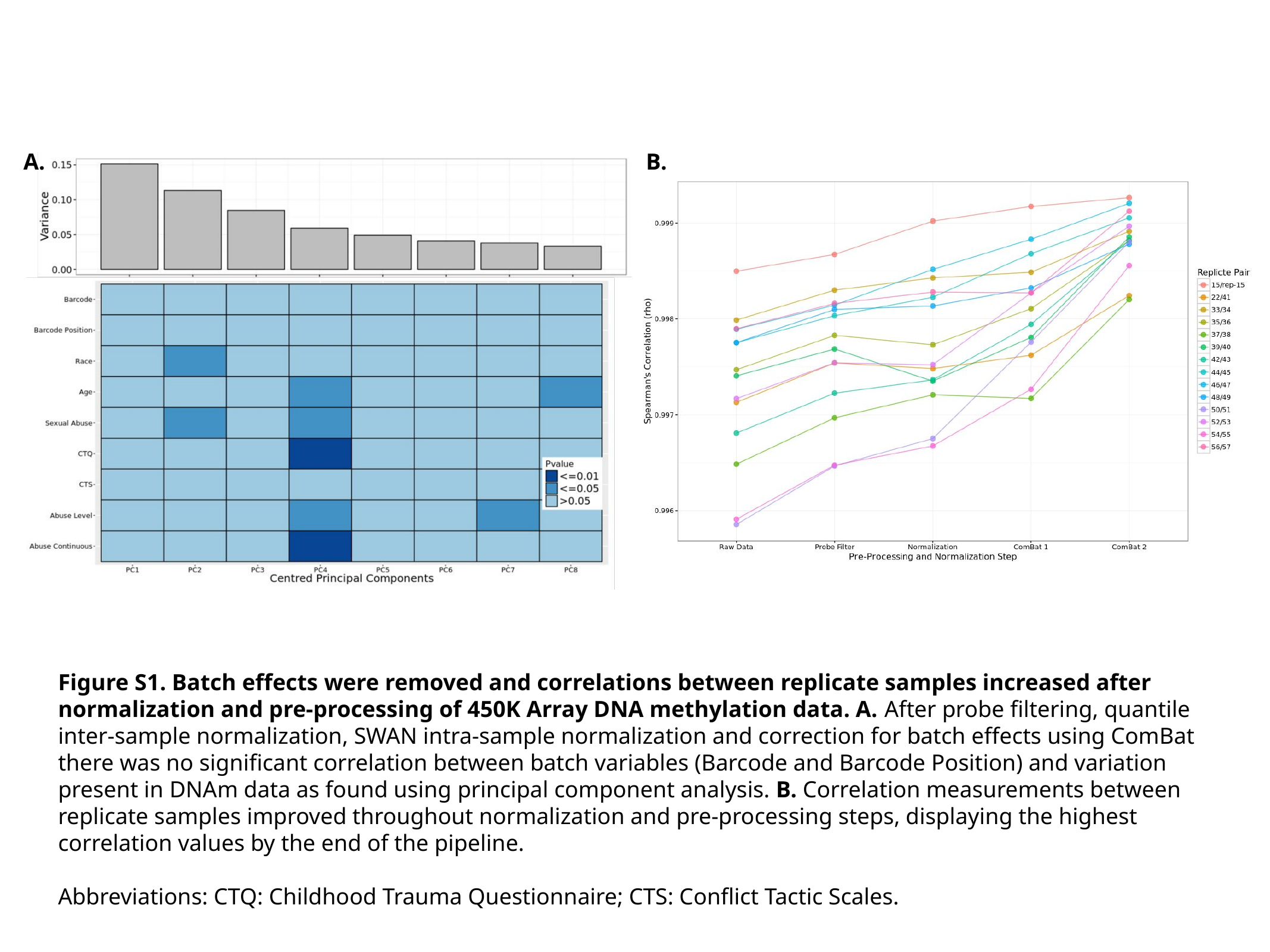

A.
B.
Figure S1. Batch effects were removed and correlations between replicate samples increased after normalization and pre-processing of 450K Array DNA methylation data. A. After probe filtering, quantile inter-sample normalization, SWAN intra-sample normalization and correction for batch effects using ComBat there was no significant correlation between batch variables (Barcode and Barcode Position) and variation present in DNAm data as found using principal component analysis. B. Correlation measurements between replicate samples improved throughout normalization and pre-processing steps, displaying the highest correlation values by the end of the pipeline.
Abbreviations: CTQ: Childhood Trauma Questionnaire; CTS: Conflict Tactic Scales.

## Slide 2
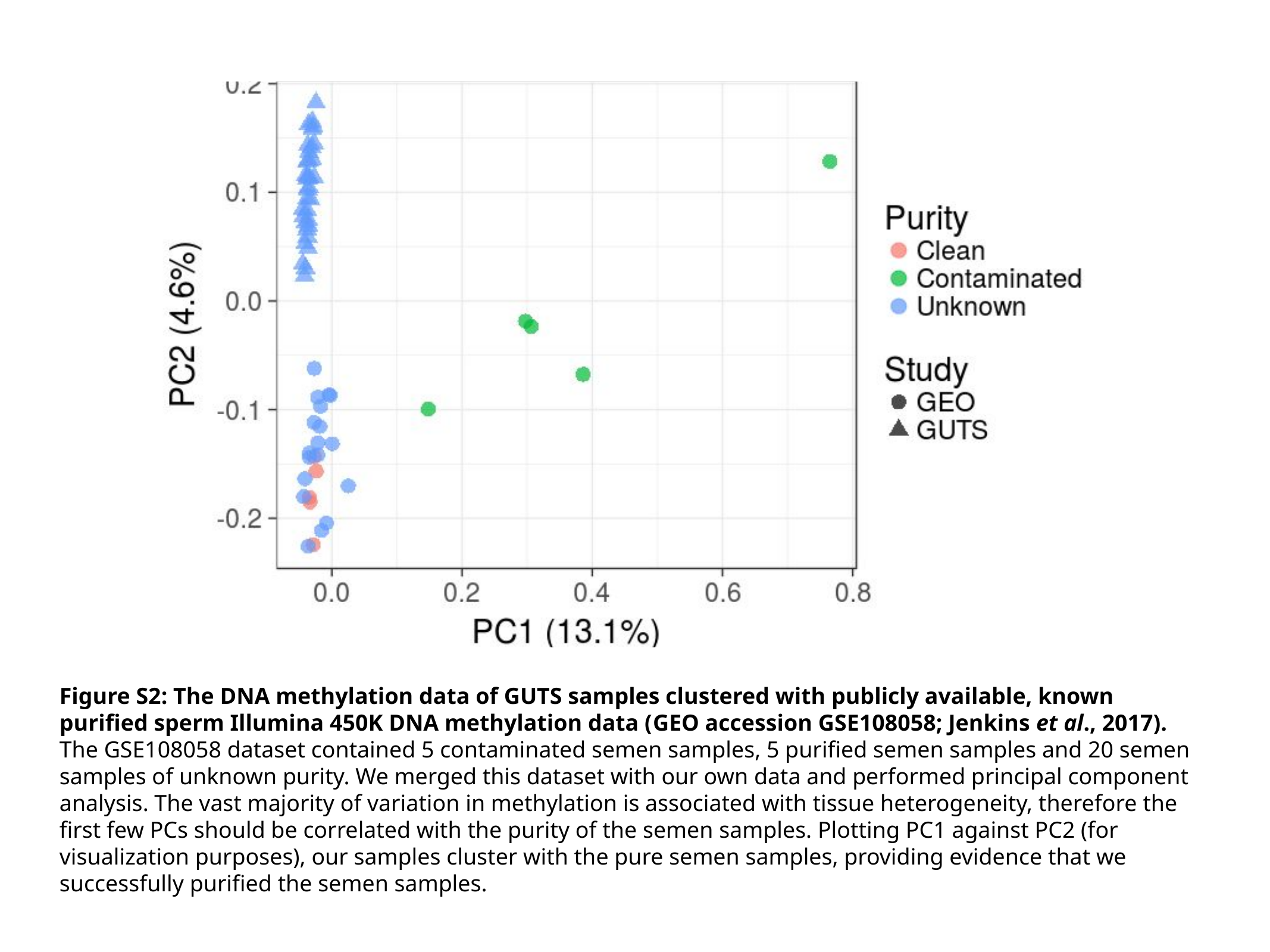

Figure S2: The DNA methylation data of GUTS samples clustered with publicly available, known purified sperm Illumina 450K DNA methylation data (GEO accession GSE108058; Jenkins et al., 2017). The GSE108058 dataset contained 5 contaminated semen samples, 5 purified semen samples and 20 semen samples of unknown purity. We merged this dataset with our own data and performed principal component analysis. The vast majority of variation in methylation is associated with tissue heterogeneity, therefore the first few PCs should be correlated with the purity of the semen samples. Plotting PC1 against PC2 (for visualization purposes), our samples cluster with the pure semen samples, providing evidence that we successfully purified the semen samples.

## Slide 3
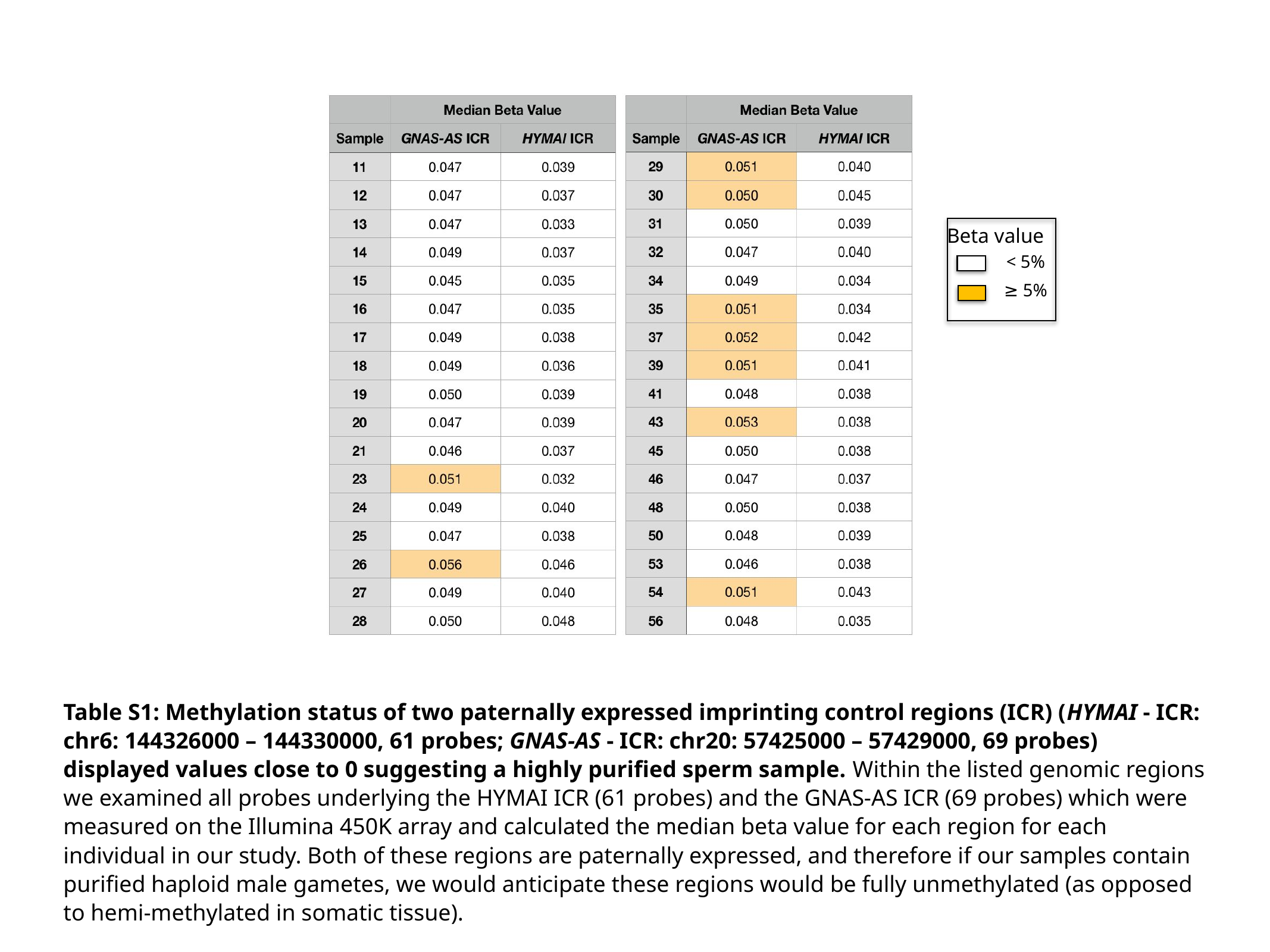

Beta value
< 5%
≥ 5%
Table S1: Methylation status of two paternally expressed imprinting control regions (ICR) (HYMAI - ICR: chr6: 144326000 – 144330000, 61 probes; GNAS-AS - ICR: chr20: 57425000 – 57429000, 69 probes) displayed values close to 0 suggesting a highly purified sperm sample. Within the listed genomic regions we examined all probes underlying the HYMAI ICR (61 probes) and the GNAS-AS ICR (69 probes) which were measured on the Illumina 450K array and calculated the median beta value for each region for each individual in our study. Both of these regions are paternally expressed, and therefore if our samples contain purified haploid male gametes, we would anticipate these regions would be fully unmethylated (as opposed to hemi-methylated in somatic tissue).

## Slide 4
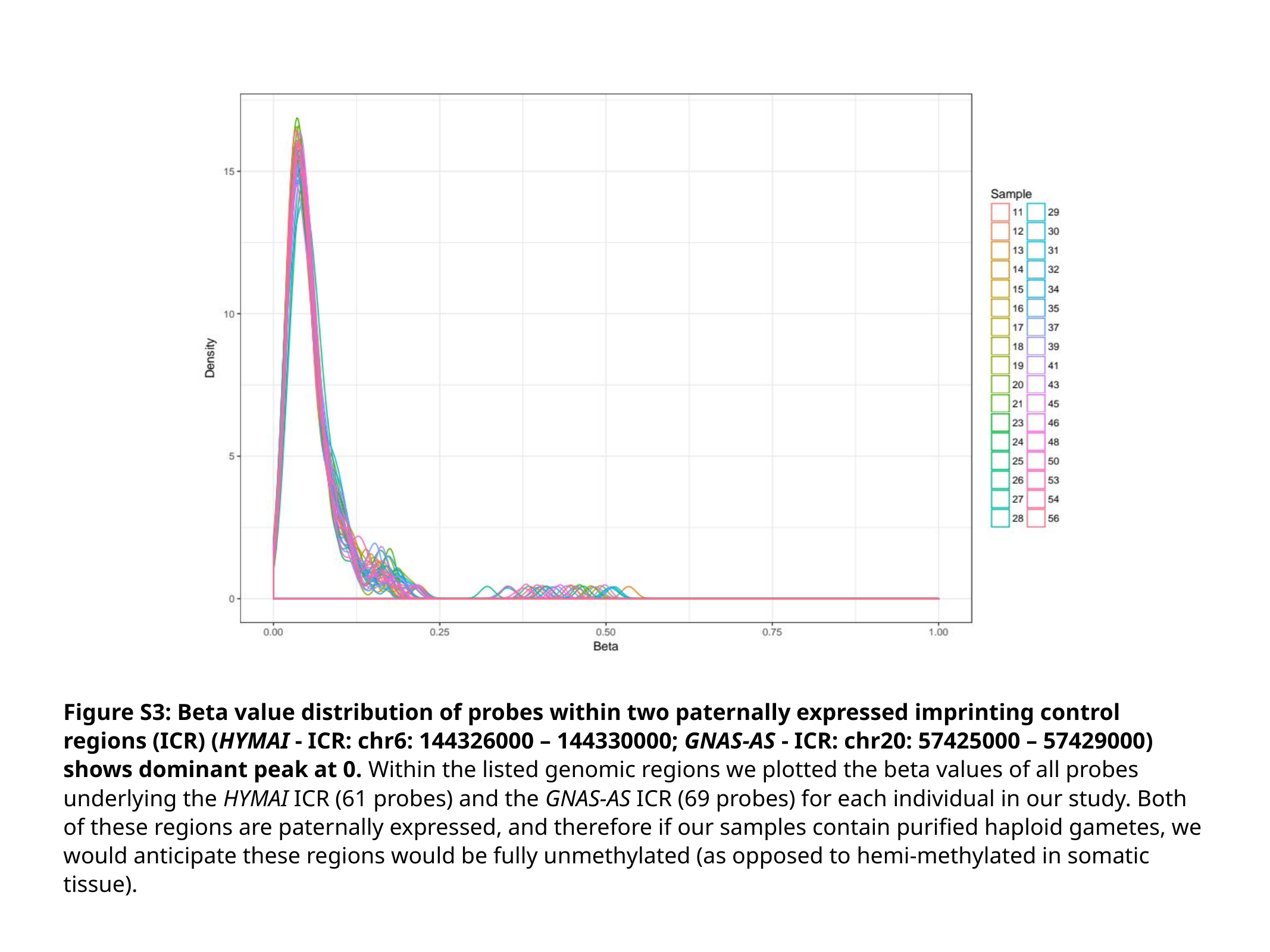

Figure S3: Beta value distribution of probes within two paternally expressed imprinting control regions (ICR) (HYMAI - ICR: chr6: 144326000 – 144330000; GNAS-AS - ICR: chr20: 57425000 – 57429000) shows dominant peak at 0. Within the listed genomic regions we plotted the beta values of all probes underlying the HYMAI ICR (61 probes) and the GNAS-AS ICR (69 probes) for each individual in our study. Both of these regions are paternally expressed, and therefore if our samples contain purified haploid gametes, we would anticipate these regions would be fully unmethylated (as opposed to hemi-methylated in somatic tissue).

## Slide 5
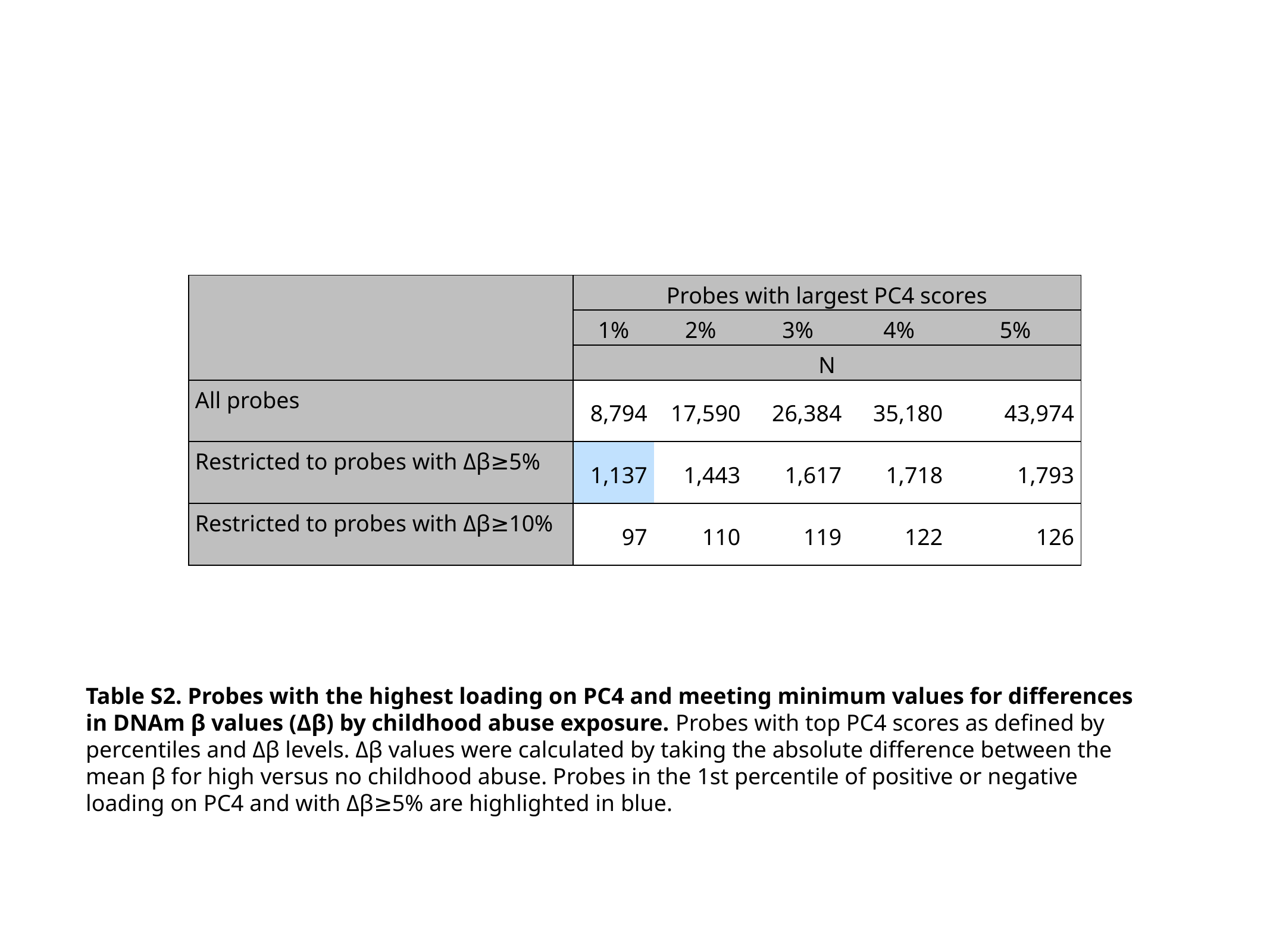

| | Probes with largest PC4 scores | | | | |
| --- | --- | --- | --- | --- | --- |
| | 1% | 2% | 3% | 4% | 5% |
| | N | | | | |
| All probes | 8,794 | 17,590 | 26,384 | 35,180 | 43,974 |
| Restricted to probes with Δβ≥5% | 1,137 | 1,443 | 1,617 | 1,718 | 1,793 |
| Restricted to probes with Δβ≥10% | 97 | 110 | 119 | 122 | 126 |
Table S2. Probes with the highest loading on PC4 and meeting minimum values for differences in DNAm β values (Δβ) by childhood abuse exposure. Probes with top PC4 scores as defined by percentiles and Δβ levels. Δβ values were calculated by taking the absolute difference between the mean β for high versus no childhood abuse. Probes in the 1st percentile of positive or negative loading on PC4 and with Δβ≥5% are highlighted in blue.

## Slide 6
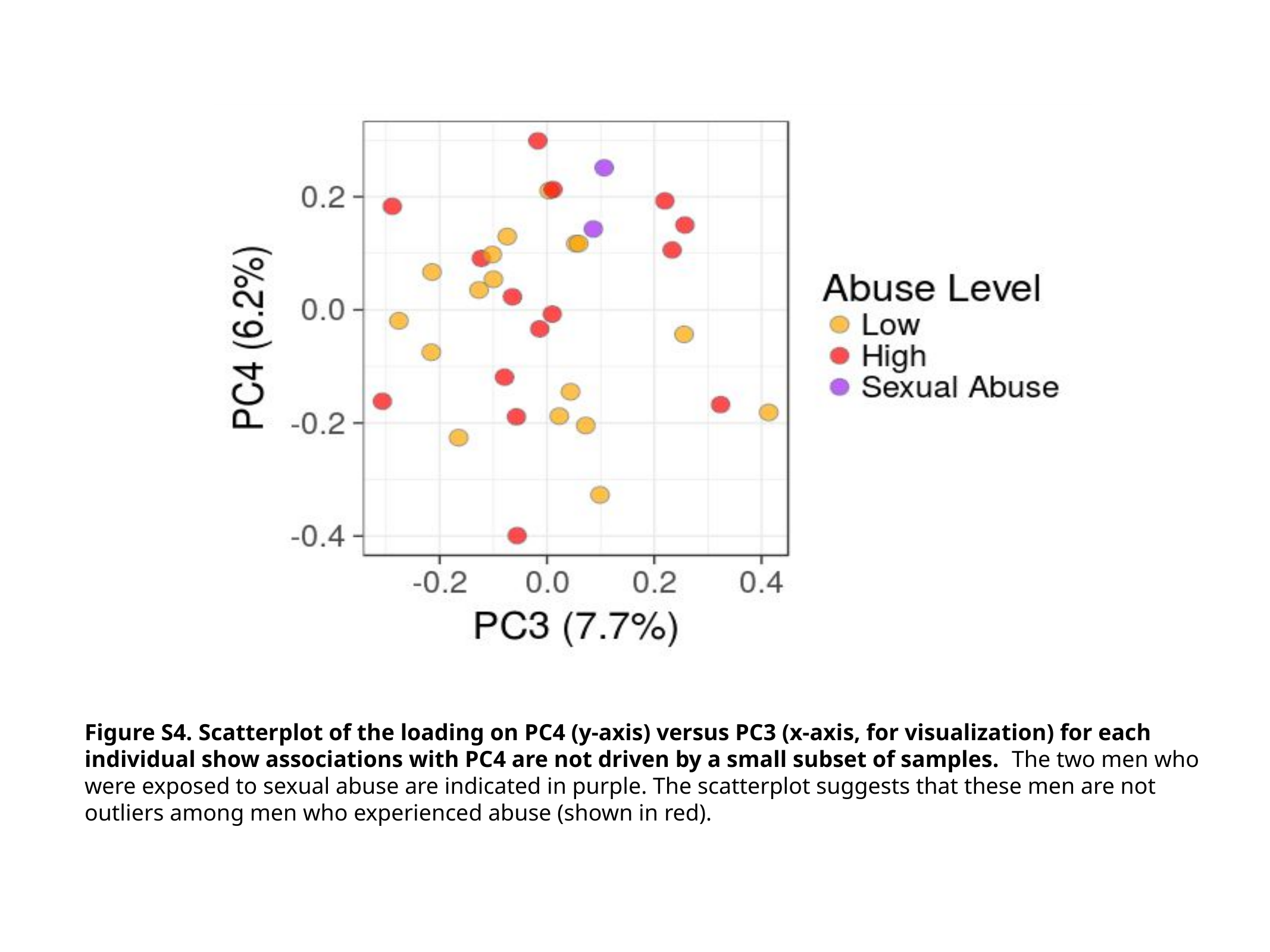

Figure S4. Scatterplot of the loading on PC4 (y-axis) versus PC3 (x-axis, for visualization) for each individual show associations with PC4 are not driven by a small subset of samples. The two men who were exposed to sexual abuse are indicated in purple. The scatterplot suggests that these men are not outliers among men who experienced abuse (shown in red).

## Slide 7
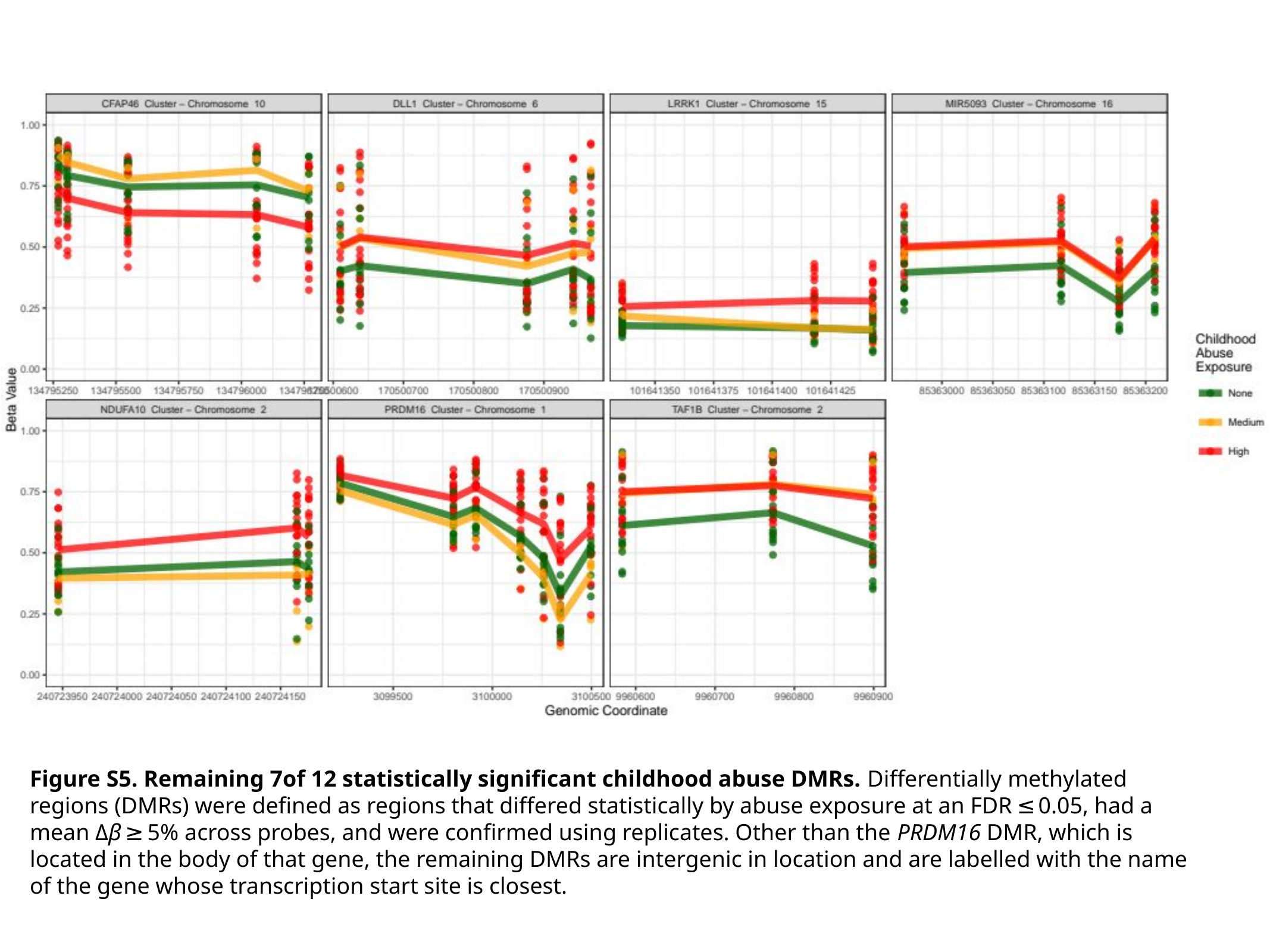

Figure S5. Remaining 7of 12 statistically significant childhood abuse DMRs. Differentially methylated regions (DMRs) were defined as regions that differed statistically by abuse exposure at an FDR ≤ 0.05, had a mean Δβ ≥ 5% across probes, and were confirmed using replicates. Other than the PRDM16 DMR, which is located in the body of that gene, the remaining DMRs are intergenic in location and are labelled with the name of the gene whose transcription start site is closest.

## Slide 8
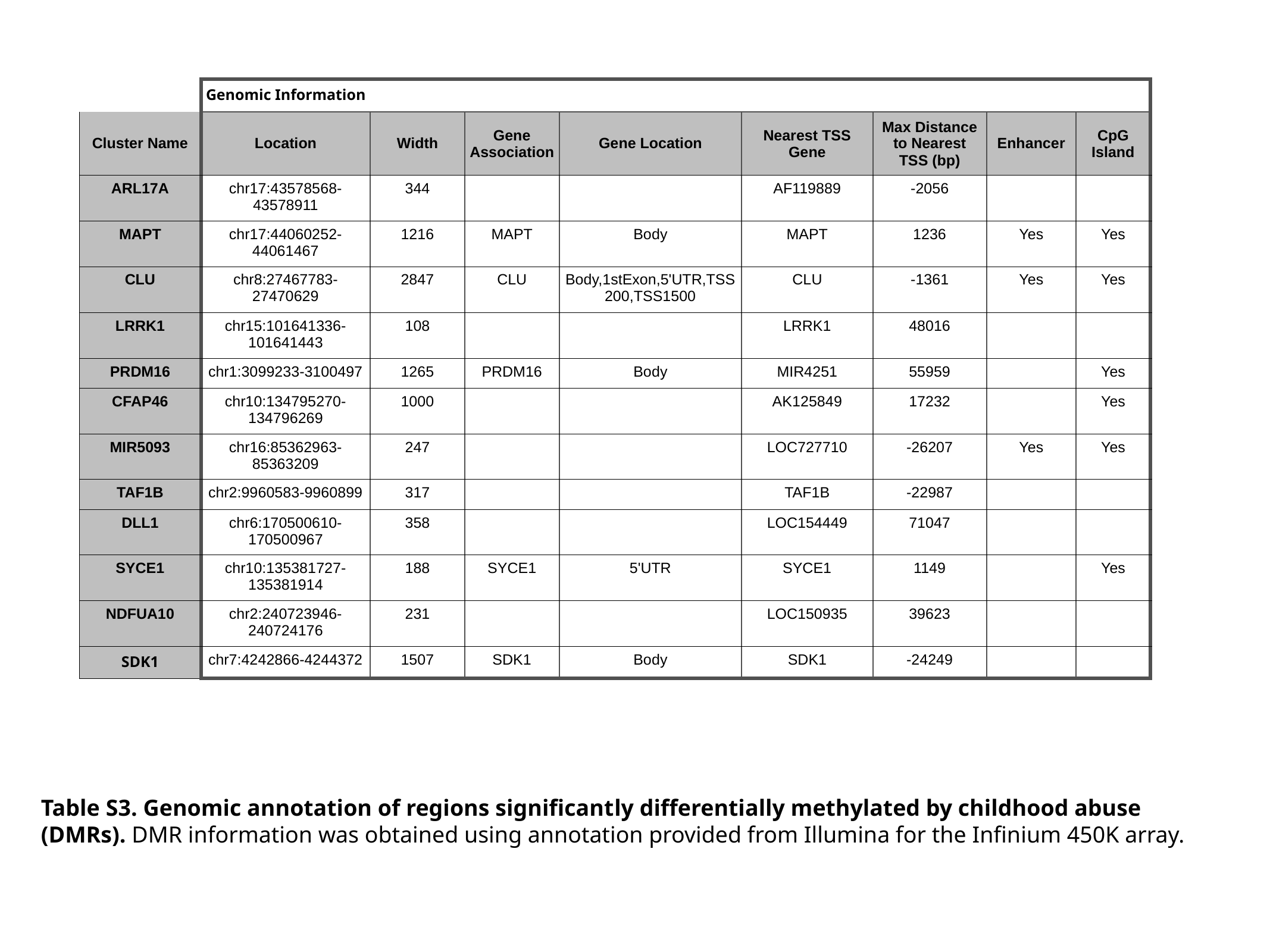

| | Genomic Information | | | | | | | |
| --- | --- | --- | --- | --- | --- | --- | --- | --- |
| Cluster Name | Location | Width | Gene Association | Gene Location | Nearest TSS Gene | Max Distance to Nearest TSS (bp) | Enhancer | CpG Island |
| ARL17A | chr17:43578568-43578911 | 344 | | | AF119889 | -2056 | | |
| MAPT | chr17:44060252-44061467 | 1216 | MAPT | Body | MAPT | 1236 | Yes | Yes |
| CLU | chr8:27467783-27470629 | 2847 | CLU | Body,1stExon,5'UTR,TSS200,TSS1500 | CLU | -1361 | Yes | Yes |
| LRRK1 | chr15:101641336-101641443 | 108 | | | LRRK1 | 48016 | | |
| PRDM16 | chr1:3099233-3100497 | 1265 | PRDM16 | Body | MIR4251 | 55959 | | Yes |
| CFAP46 | chr10:134795270-134796269 | 1000 | | | AK125849 | 17232 | | Yes |
| MIR5093 | chr16:85362963-85363209 | 247 | | | LOC727710 | -26207 | Yes | Yes |
| TAF1B | chr2:9960583-9960899 | 317 | | | TAF1B | -22987 | | |
| DLL1 | chr6:170500610-170500967 | 358 | | | LOC154449 | 71047 | | |
| SYCE1 | chr10:135381727-135381914 | 188 | SYCE1 | 5'UTR | SYCE1 | 1149 | | Yes |
| NDFUA10 | chr2:240723946-240724176 | 231 | | | LOC150935 | 39623 | | |
| SDK1 | chr7:4242866-4244372 | 1507 | SDK1 | Body | SDK1 | -24249 | | |
Table S3. Genomic annotation of regions significantly differentially methylated by childhood abuse (DMRs). DMR information was obtained using annotation provided from Illumina for the Infinium 450K array.

## Slide 9
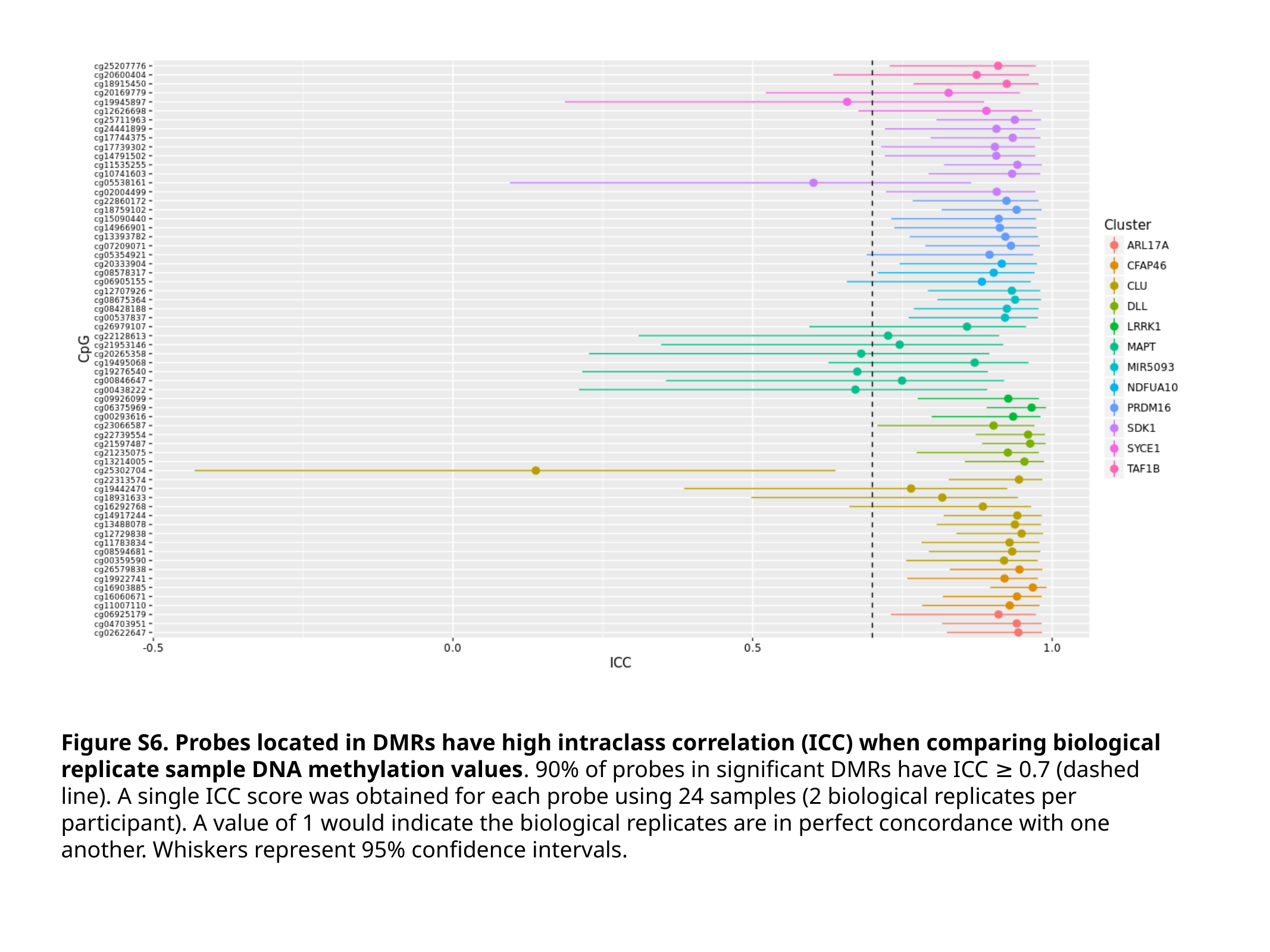

Figure S6. Probes located in DMRs have high intraclass correlation (ICC) when comparing biological replicate sample DNA methylation values. 90% of probes in significant DMRs have ICC ≥ 0.7 (dashed line). A single ICC score was obtained for each probe using 24 samples (2 biological replicates per participant). A value of 1 would indicate the biological replicates are in perfect concordance with one another. Whiskers represent 95% confidence intervals.

## Slide 10
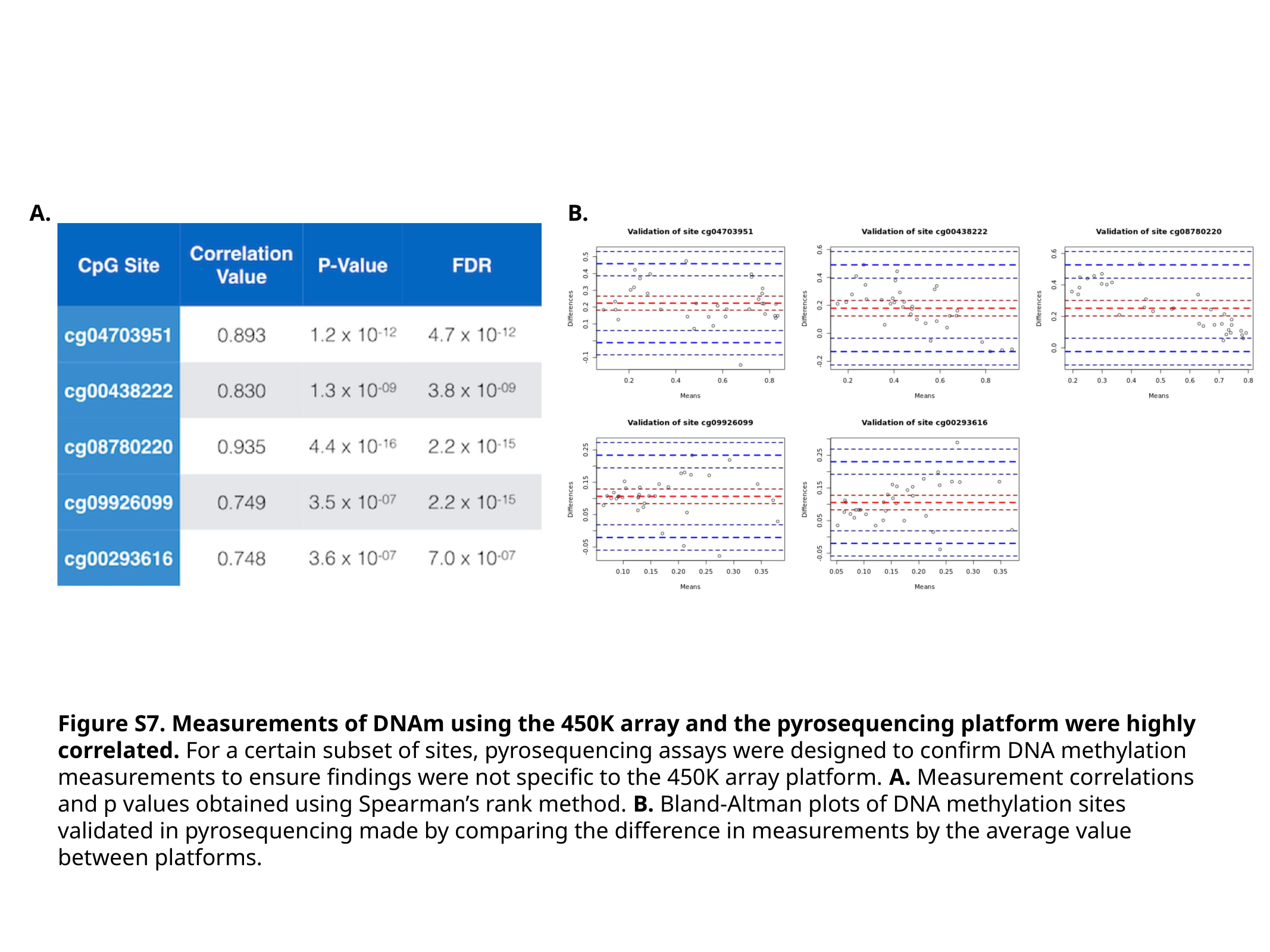

A.
B.
Figure S7. Measurements of DNAm using the 450K array and the pyrosequencing platform were highly correlated. For a certain subset of sites, pyrosequencing assays were designed to confirm DNA methylation measurements to ensure findings were not specific to the 450K array platform. A. Measurement correlations and p values obtained using Spearman’s rank method. B. Bland-Altman plots of DNA methylation sites validated in pyrosequencing made by comparing the difference in measurements by the average value between platforms.

## Slide 11
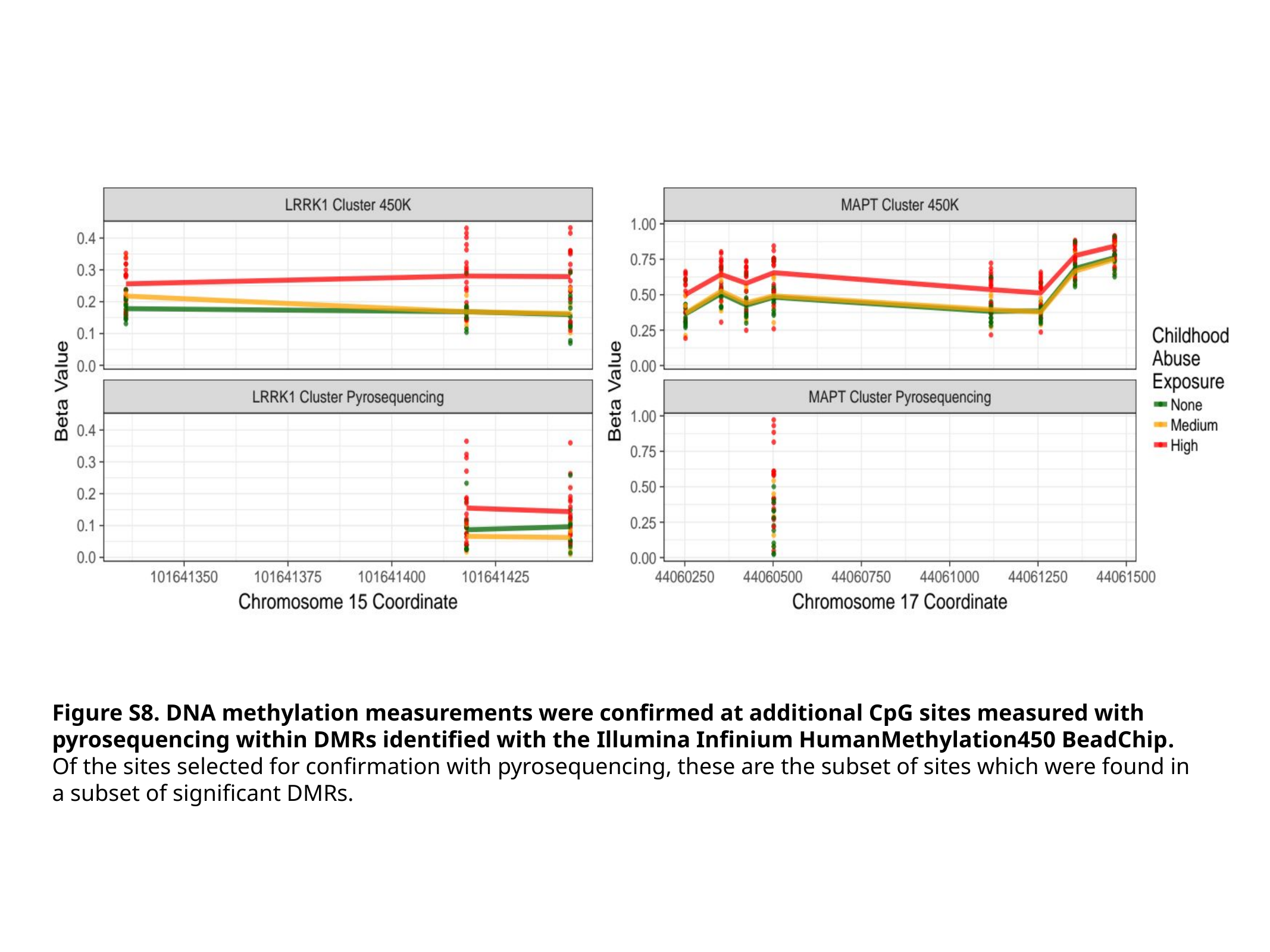

Figure S8. DNA methylation measurements were confirmed at additional CpG sites measured with pyrosequencing within DMRs identified with the Illumina Infinium HumanMethylation450 BeadChip. Of the sites selected for confirmation with pyrosequencing, these are the subset of sites which were found in a subset of significant DMRs.

## Slide 12
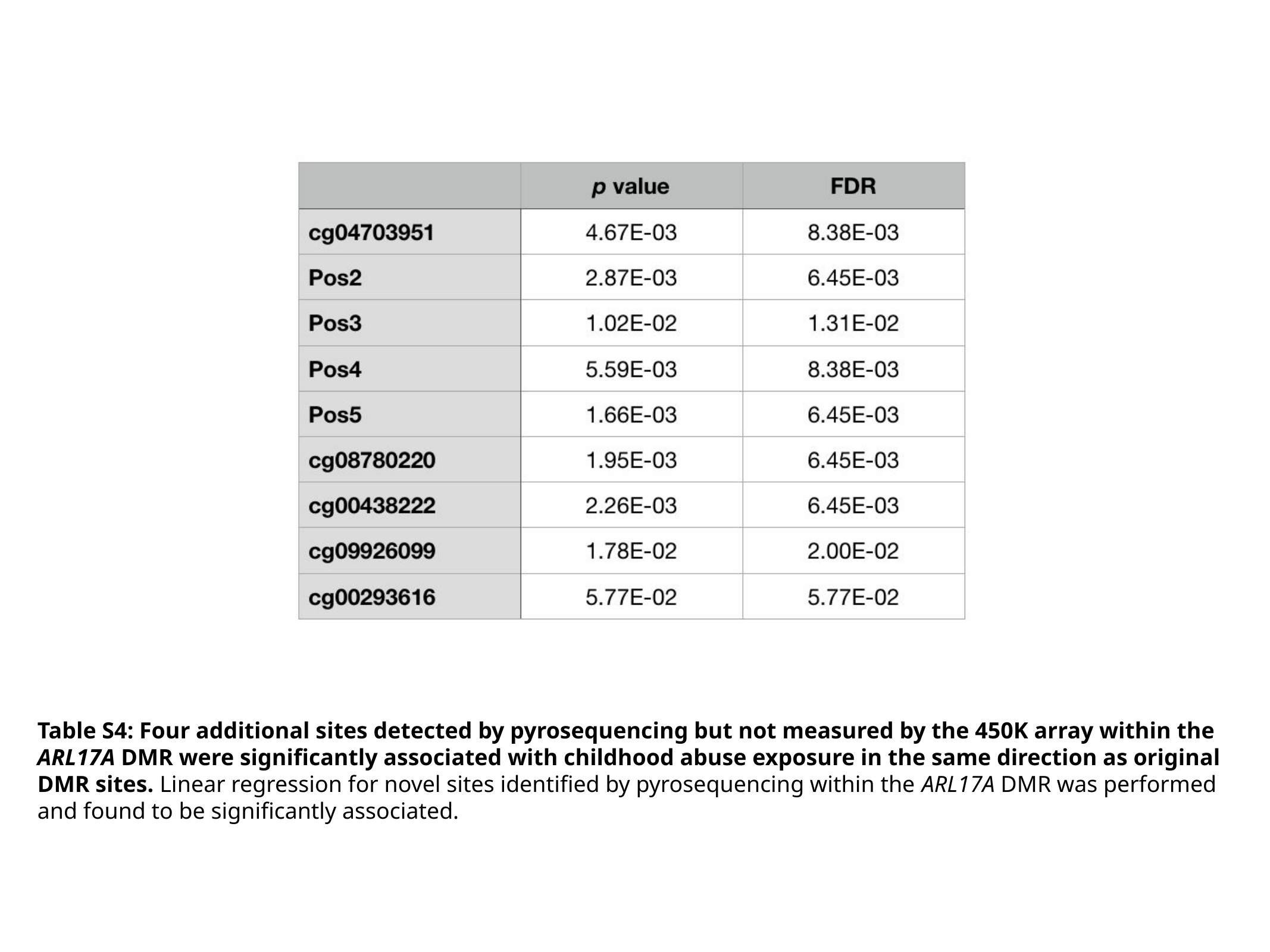

Table S4: Four additional sites detected by pyrosequencing but not measured by the 450K array within the ARL17A DMR were significantly associated with childhood abuse exposure in the same direction as original DMR sites. Linear regression for novel sites identified by pyrosequencing within the ARL17A DMR was performed and found to be significantly associated.

## Slide 13
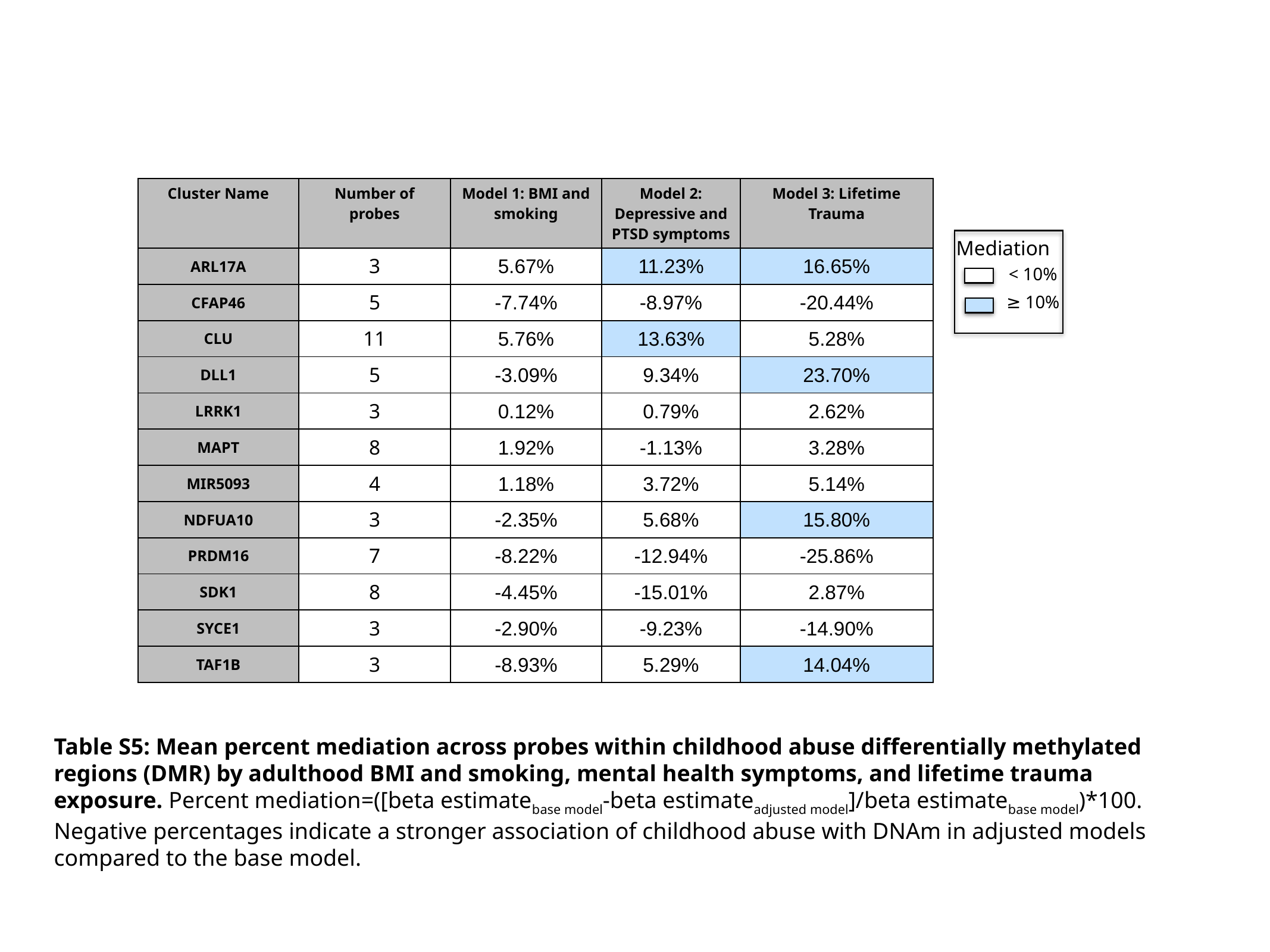

| Cluster Name | Number of probes | Model 1: BMI and smoking | Model 2: Depressive and PTSD symptoms | Model 3: Lifetime Trauma |
| --- | --- | --- | --- | --- |
| ARL17A | 3 | 5.67% | 11.23% | 16.65% |
| CFAP46 | 5 | -7.74% | -8.97% | -20.44% |
| CLU | 11 | 5.76% | 13.63% | 5.28% |
| DLL1 | 5 | -3.09% | 9.34% | 23.70% |
| LRRK1 | 3 | 0.12% | 0.79% | 2.62% |
| MAPT | 8 | 1.92% | -1.13% | 3.28% |
| MIR5093 | 4 | 1.18% | 3.72% | 5.14% |
| NDFUA10 | 3 | -2.35% | 5.68% | 15.80% |
| PRDM16 | 7 | -8.22% | -12.94% | -25.86% |
| SDK1 | 8 | -4.45% | -15.01% | 2.87% |
| SYCE1 | 3 | -2.90% | -9.23% | -14.90% |
| TAF1B | 3 | -8.93% | 5.29% | 14.04% |
Mediation
< 10%
≥ 10%
Table S5: Mean percent mediation across probes within childhood abuse differentially methylated regions (DMR) by adulthood BMI and smoking, mental health symptoms, and lifetime trauma exposure. Percent mediation=([beta estimatebase model-beta estimateadjusted model]/beta estimatebase model)*100. Negative percentages indicate a stronger association of childhood abuse with DNAm in adjusted models compared to the base model.

## Slide 14
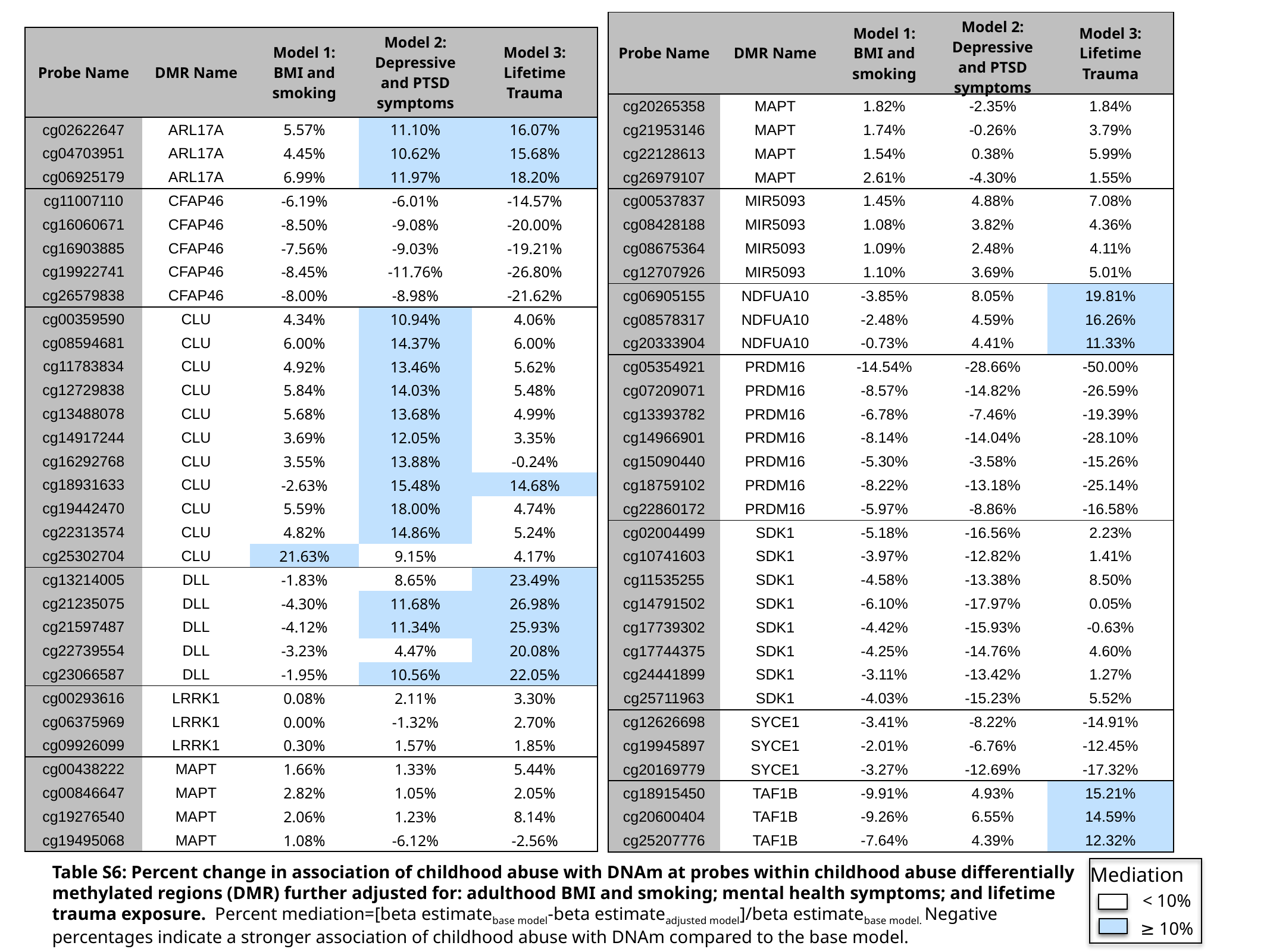

| Probe Name | DMR Name | Model 1: BMI and smoking | Model 2: Depressive and PTSD symptoms | Model 3: Lifetime Trauma |
| --- | --- | --- | --- | --- |
| cg20265358 | MAPT | 1.82% | -2.35% | 1.84% |
| cg21953146 | MAPT | 1.74% | -0.26% | 3.79% |
| cg22128613 | MAPT | 1.54% | 0.38% | 5.99% |
| cg26979107 | MAPT | 2.61% | -4.30% | 1.55% |
| cg00537837 | MIR5093 | 1.45% | 4.88% | 7.08% |
| cg08428188 | MIR5093 | 1.08% | 3.82% | 4.36% |
| cg08675364 | MIR5093 | 1.09% | 2.48% | 4.11% |
| cg12707926 | MIR5093 | 1.10% | 3.69% | 5.01% |
| cg06905155 | NDFUA10 | -3.85% | 8.05% | 19.81% |
| cg08578317 | NDFUA10 | -2.48% | 4.59% | 16.26% |
| cg20333904 | NDFUA10 | -0.73% | 4.41% | 11.33% |
| cg05354921 | PRDM16 | -14.54% | -28.66% | -50.00% |
| cg07209071 | PRDM16 | -8.57% | -14.82% | -26.59% |
| cg13393782 | PRDM16 | -6.78% | -7.46% | -19.39% |
| cg14966901 | PRDM16 | -8.14% | -14.04% | -28.10% |
| cg15090440 | PRDM16 | -5.30% | -3.58% | -15.26% |
| cg18759102 | PRDM16 | -8.22% | -13.18% | -25.14% |
| cg22860172 | PRDM16 | -5.97% | -8.86% | -16.58% |
| cg02004499 | SDK1 | -5.18% | -16.56% | 2.23% |
| cg10741603 | SDK1 | -3.97% | -12.82% | 1.41% |
| cg11535255 | SDK1 | -4.58% | -13.38% | 8.50% |
| cg14791502 | SDK1 | -6.10% | -17.97% | 0.05% |
| cg17739302 | SDK1 | -4.42% | -15.93% | -0.63% |
| cg17744375 | SDK1 | -4.25% | -14.76% | 4.60% |
| cg24441899 | SDK1 | -3.11% | -13.42% | 1.27% |
| cg25711963 | SDK1 | -4.03% | -15.23% | 5.52% |
| cg12626698 | SYCE1 | -3.41% | -8.22% | -14.91% |
| cg19945897 | SYCE1 | -2.01% | -6.76% | -12.45% |
| cg20169779 | SYCE1 | -3.27% | -12.69% | -17.32% |
| cg18915450 | TAF1B | -9.91% | 4.93% | 15.21% |
| cg20600404 | TAF1B | -9.26% | 6.55% | 14.59% |
| cg25207776 | TAF1B | -7.64% | 4.39% | 12.32% |
| Probe Name | DMR Name | Model 1: BMI and smoking | Model 2: Depressive and PTSD symptoms | Model 3: Lifetime Trauma |
| --- | --- | --- | --- | --- |
| cg02622647 | ARL17A | 5.57% | 11.10% | 16.07% |
| cg04703951 | ARL17A | 4.45% | 10.62% | 15.68% |
| cg06925179 | ARL17A | 6.99% | 11.97% | 18.20% |
| cg11007110 | CFAP46 | -6.19% | -6.01% | -14.57% |
| cg16060671 | CFAP46 | -8.50% | -9.08% | -20.00% |
| cg16903885 | CFAP46 | -7.56% | -9.03% | -19.21% |
| cg19922741 | CFAP46 | -8.45% | -11.76% | -26.80% |
| cg26579838 | CFAP46 | -8.00% | -8.98% | -21.62% |
| cg00359590 | CLU | 4.34% | 10.94% | 4.06% |
| cg08594681 | CLU | 6.00% | 14.37% | 6.00% |
| cg11783834 | CLU | 4.92% | 13.46% | 5.62% |
| cg12729838 | CLU | 5.84% | 14.03% | 5.48% |
| cg13488078 | CLU | 5.68% | 13.68% | 4.99% |
| cg14917244 | CLU | 3.69% | 12.05% | 3.35% |
| cg16292768 | CLU | 3.55% | 13.88% | -0.24% |
| cg18931633 | CLU | -2.63% | 15.48% | 14.68% |
| cg19442470 | CLU | 5.59% | 18.00% | 4.74% |
| cg22313574 | CLU | 4.82% | 14.86% | 5.24% |
| cg25302704 | CLU | 21.63% | 9.15% | 4.17% |
| cg13214005 | DLL | -1.83% | 8.65% | 23.49% |
| cg21235075 | DLL | -4.30% | 11.68% | 26.98% |
| cg21597487 | DLL | -4.12% | 11.34% | 25.93% |
| cg22739554 | DLL | -3.23% | 4.47% | 20.08% |
| cg23066587 | DLL | -1.95% | 10.56% | 22.05% |
| cg00293616 | LRRK1 | 0.08% | 2.11% | 3.30% |
| cg06375969 | LRRK1 | 0.00% | -1.32% | 2.70% |
| cg09926099 | LRRK1 | 0.30% | 1.57% | 1.85% |
| cg00438222 | MAPT | 1.66% | 1.33% | 5.44% |
| cg00846647 | MAPT | 2.82% | 1.05% | 2.05% |
| cg19276540 | MAPT | 2.06% | 1.23% | 8.14% |
| cg19495068 | MAPT | 1.08% | -6.12% | -2.56% |
Table S6: Percent change in association of childhood abuse with DNAm at probes within childhood abuse differentially methylated regions (DMR) further adjusted for: adulthood BMI and smoking; mental health symptoms; and lifetime trauma exposure. Percent mediation=[beta estimatebase model-beta estimateadjusted model]/beta estimatebase model. Negative percentages indicate a stronger association of childhood abuse with DNAm compared to the base model.
Mediation
< 10%
≥ 10%

## Slide 15
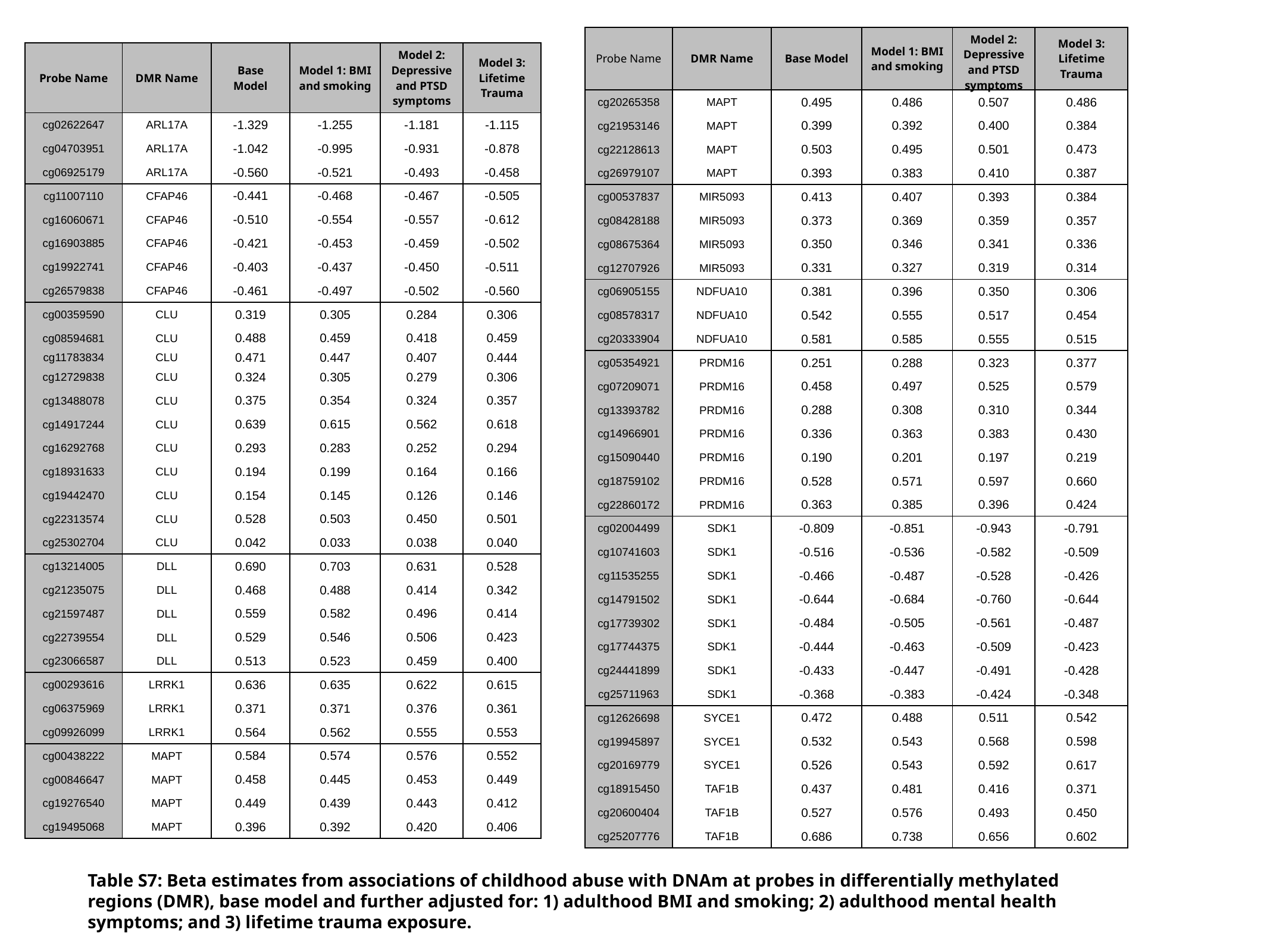

| Probe Name | DMR Name | Base Model | Model 1: BMI and smoking | Model 2: Depressive and PTSD symptoms | Model 3: Lifetime Trauma |
| --- | --- | --- | --- | --- | --- |
| cg20265358 | MAPT | 0.495 | 0.486 | 0.507 | 0.486 |
| cg21953146 | MAPT | 0.399 | 0.392 | 0.400 | 0.384 |
| cg22128613 | MAPT | 0.503 | 0.495 | 0.501 | 0.473 |
| cg26979107 | MAPT | 0.393 | 0.383 | 0.410 | 0.387 |
| cg00537837 | MIR5093 | 0.413 | 0.407 | 0.393 | 0.384 |
| cg08428188 | MIR5093 | 0.373 | 0.369 | 0.359 | 0.357 |
| cg08675364 | MIR5093 | 0.350 | 0.346 | 0.341 | 0.336 |
| cg12707926 | MIR5093 | 0.331 | 0.327 | 0.319 | 0.314 |
| cg06905155 | NDFUA10 | 0.381 | 0.396 | 0.350 | 0.306 |
| cg08578317 | NDFUA10 | 0.542 | 0.555 | 0.517 | 0.454 |
| cg20333904 | NDFUA10 | 0.581 | 0.585 | 0.555 | 0.515 |
| cg05354921 | PRDM16 | 0.251 | 0.288 | 0.323 | 0.377 |
| cg07209071 | PRDM16 | 0.458 | 0.497 | 0.525 | 0.579 |
| cg13393782 | PRDM16 | 0.288 | 0.308 | 0.310 | 0.344 |
| cg14966901 | PRDM16 | 0.336 | 0.363 | 0.383 | 0.430 |
| cg15090440 | PRDM16 | 0.190 | 0.201 | 0.197 | 0.219 |
| cg18759102 | PRDM16 | 0.528 | 0.571 | 0.597 | 0.660 |
| cg22860172 | PRDM16 | 0.363 | 0.385 | 0.396 | 0.424 |
| cg02004499 | SDK1 | -0.809 | -0.851 | -0.943 | -0.791 |
| cg10741603 | SDK1 | -0.516 | -0.536 | -0.582 | -0.509 |
| cg11535255 | SDK1 | -0.466 | -0.487 | -0.528 | -0.426 |
| cg14791502 | SDK1 | -0.644 | -0.684 | -0.760 | -0.644 |
| cg17739302 | SDK1 | -0.484 | -0.505 | -0.561 | -0.487 |
| cg17744375 | SDK1 | -0.444 | -0.463 | -0.509 | -0.423 |
| cg24441899 | SDK1 | -0.433 | -0.447 | -0.491 | -0.428 |
| cg25711963 | SDK1 | -0.368 | -0.383 | -0.424 | -0.348 |
| cg12626698 | SYCE1 | 0.472 | 0.488 | 0.511 | 0.542 |
| cg19945897 | SYCE1 | 0.532 | 0.543 | 0.568 | 0.598 |
| cg20169779 | SYCE1 | 0.526 | 0.543 | 0.592 | 0.617 |
| cg18915450 | TAF1B | 0.437 | 0.481 | 0.416 | 0.371 |
| cg20600404 | TAF1B | 0.527 | 0.576 | 0.493 | 0.450 |
| cg25207776 | TAF1B | 0.686 | 0.738 | 0.656 | 0.602 |
| Probe Name | DMR Name | Base Model | Model 1: BMI and smoking | Model 2: Depressive and PTSD symptoms | Model 3: Lifetime Trauma |
| --- | --- | --- | --- | --- | --- |
| cg02622647 | ARL17A | -1.329 | -1.255 | -1.181 | -1.115 |
| cg04703951 | ARL17A | -1.042 | -0.995 | -0.931 | -0.878 |
| cg06925179 | ARL17A | -0.560 | -0.521 | -0.493 | -0.458 |
| cg11007110 | CFAP46 | -0.441 | -0.468 | -0.467 | -0.505 |
| cg16060671 | CFAP46 | -0.510 | -0.554 | -0.557 | -0.612 |
| cg16903885 | CFAP46 | -0.421 | -0.453 | -0.459 | -0.502 |
| cg19922741 | CFAP46 | -0.403 | -0.437 | -0.450 | -0.511 |
| cg26579838 | CFAP46 | -0.461 | -0.497 | -0.502 | -0.560 |
| cg00359590 | CLU | 0.319 | 0.305 | 0.284 | 0.306 |
| cg08594681 | CLU | 0.488 | 0.459 | 0.418 | 0.459 |
| cg11783834 | CLU | 0.471 | 0.447 | 0.407 | 0.444 |
| cg12729838 | CLU | 0.324 | 0.305 | 0.279 | 0.306 |
| cg13488078 | CLU | 0.375 | 0.354 | 0.324 | 0.357 |
| cg14917244 | CLU | 0.639 | 0.615 | 0.562 | 0.618 |
| cg16292768 | CLU | 0.293 | 0.283 | 0.252 | 0.294 |
| cg18931633 | CLU | 0.194 | 0.199 | 0.164 | 0.166 |
| cg19442470 | CLU | 0.154 | 0.145 | 0.126 | 0.146 |
| cg22313574 | CLU | 0.528 | 0.503 | 0.450 | 0.501 |
| cg25302704 | CLU | 0.042 | 0.033 | 0.038 | 0.040 |
| cg13214005 | DLL | 0.690 | 0.703 | 0.631 | 0.528 |
| cg21235075 | DLL | 0.468 | 0.488 | 0.414 | 0.342 |
| cg21597487 | DLL | 0.559 | 0.582 | 0.496 | 0.414 |
| cg22739554 | DLL | 0.529 | 0.546 | 0.506 | 0.423 |
| cg23066587 | DLL | 0.513 | 0.523 | 0.459 | 0.400 |
| cg00293616 | LRRK1 | 0.636 | 0.635 | 0.622 | 0.615 |
| cg06375969 | LRRK1 | 0.371 | 0.371 | 0.376 | 0.361 |
| cg09926099 | LRRK1 | 0.564 | 0.562 | 0.555 | 0.553 |
| cg00438222 | MAPT | 0.584 | 0.574 | 0.576 | 0.552 |
| cg00846647 | MAPT | 0.458 | 0.445 | 0.453 | 0.449 |
| cg19276540 | MAPT | 0.449 | 0.439 | 0.443 | 0.412 |
| cg19495068 | MAPT | 0.396 | 0.392 | 0.420 | 0.406 |
Table S7: Beta estimates from associations of childhood abuse with DNAm at probes in differentially methylated regions (DMR), base model and further adjusted for: 1) adulthood BMI and smoking; 2) adulthood mental health symptoms; and 3) lifetime trauma exposure.
